# A map of metabolic phenotypes in patients with myalgic encephalomyelitis/chronic fatigue syndrome

**DOI:** 10.1172/jci.insight.149217

**Published:** 2021-08-23

**Authors:** Fredrik Hoel, August Hoel, Ina K.N. Pettersen, Ingrid G. Rekeland, Kristin Risa, Kine Alme, Kari Sørland, Alexander Fosså, Katarina Lien, Ingrid Herder, Hanne L. Thürmer, Merete E. Gotaas, Christoph Schäfer, Rolf K. Berge, Kristian Sommerfelt, Hans-Peter Marti, Olav Dahl, Olav Mella, Øystein Fluge, Karl J. Tronstad

**Affiliations:** 1 Department of Biomedicine and; 2 Department of Clinical Medicine, University of Bergen, Bergen, Norway.; 3 Department of Oncology and Medical Physics, Haukeland University Hospital, Bergen, Norway.; 4 Department of Oncology, Norwegian Radium Hospital, Oslo University Hospital, Oslo, Norway.; 5 KJ Jebsen Centre for B-cell malignancies, University of Oslo, Oslo, Norway.; 6 CFS/ME Center, Division of Medicine, Oslo University Hospital, Oslo, Norway.; 7 Department of Medicine, Telemark Hospital, Notodden, Norway.; 8 Department of Pain and Complex Disorders, St. Olav’s Hospital, Trondheim, Norway.; 9 Department of Rehabilitation Medicine, University Hospital of North Norway, Tromsø, Norway.; 10 Department of Clinical Science, University of Bergen, Bergen, Norway.; 11 Department of Pediatrics and; 12 Department of Medicine, Haukeland University Hospital, Bergen, Norway.

**Keywords:** Immunology, Metabolism, Bioinformatics, Fatty acid oxidation, Intermediary metabolism

## Abstract

Myalgic encephalomyelitis/chronic fatigue syndrome (ME/CFS) is a debilitating disease usually presenting after infection. Emerging evidence supports that energy metabolism is affected in ME/CFS, but a unifying metabolic phenotype has not been firmly established. We performed global metabolomics, lipidomics, and hormone measurements, and we used exploratory data analyses to compare serum from 83 patients with ME/CFS and 35 healthy controls. Some changes were common in the patient group, and these were compatible with effects of elevated energy strain and altered utilization of fatty acids and amino acids as catabolic fuels. In addition, a set of heterogeneous effects reflected specific changes in 3 subsets of patients, and 2 of these expressed characteristic contexts of deregulated energy metabolism. The biological relevance of these metabolic phenotypes (metabotypes) was supported by clinical data and independent blood analyses. In summary, we report a map of common and context-dependent metabolic changes in ME/CFS, and some of them presented possible associations with clinical patient profiles. We suggest that elevated energy strain may result from exertion-triggered tissue hypoxia and lead to systemic metabolic adaptation and compensation. Through various mechanisms, such metabolic dysfunction represents a likely mediator of key symptoms in ME/CFS and possibly a target for supportive intervention.

## Graphical abstract

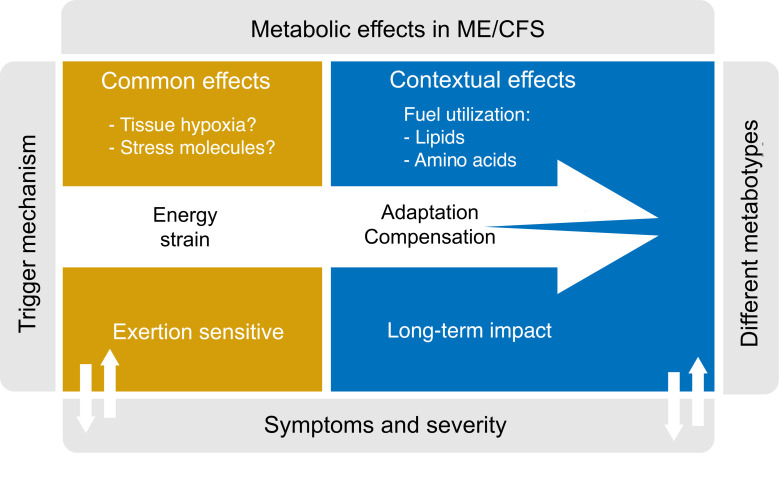

## Introduction

Myalgic encephalomyelitis/chronic fatigue syndrome (ME/CFS) typically presents with a sudden onset following an infection. Key symptoms are debilitating and long-lasting fatigue, combined with postexertional malaise causing prolonged exacerbated symptom burden after physical or mental overactivity. ME/CFS affects multiple organ systems, and additional symptoms include unrefreshing sleep, cognitive problems (referred to as brain fog), autonomic dysfunction, sensory hypersensitivity, and widespread pain (1, 2). Using the Canadian consensus criteria (1), the prevalence of ME/CFS is 0.1%–0.8% in both adults and children (3–5). Patients recovering from SARS-CoV-2 infection (COVID-19) may develop ME/CFS-like illness (“long COVID”; refs. 6, 7). While the etiology of ME/CFS is still unknown, accumulating evidence documents measurable biological changes in the blood of patients with ME/CFS. A deeper understanding of the underlying biological mechanisms is urgently required to meet this major health challenge.

ME/CFS has been suggested to be triggered by an aberrant immune response, with possible roles for autoimmunity, immune dysregulation, and inflammation (8–13). Pathological effects reported in the patients include neuroinflammation, neuroendocrine abnormalities, autonomic nervous system abnormalities, disturbed energy metabolism, and immunological changes (14). Such effects are inherently connected through multifaceted physiological cues at molecular, cellular, and systemic levels. We hypothesize that impaired metabolism and strain on cellular energetics may play a central role. Functional evidence supports the existence of ME/CFS serum factors that stress cell metabolism in vitro (15–17). Despite the fact that metabolic blood parameters usually are within the normal ranges in patients with ME/CFS (18), studies have indicated subclinical abnormalities and metabolic shifts that may have biological relevance. For example, significant or trending changes in blood glucose and lipids have been reported (19–23). Such effects may suggest that systemic control of energy fuel storage, mobilization, and utilization is affected in patients with ME/CFS, but a consensus phenotype regarding energy metabolism has not been established yet.

Untargeted metabolomics studies have found alterations that associate with the disease (20, 21, 24–26). The reported metabolite patterns are generally compatible with disturbed cellular energetics, although the affected metabolites vary somewhat between the studies. Furthermore, the relatively large data heterogeneity often seen within ME/CFS cohorts may indicate the presence of context-dependent individual effects. However, the notion that impaired energy metabolism is implicated in ME/CFS is further supported by targeted high-confidence studies that show changes related to key pathways such as the tricarboxylic acid (TCA) cycle and amino acid metabolism (15, 27–29). Some metabolic signatures reported in ME/CFS appear to be common in other chronic diseases, whereas others seem more specific. For example, the reduced levels of branched chain amino acids (BCAAs) reported in patients with ME/CFS (15, 26, 28) are contrary to the increased levels generally related to inactivity, obesity, and insulin resistance (30–32).

Metabolism plays a central role in inherent physiological programs that evolved to protect against deficiencies and threats, such as starvation, hypoxia, and infection (33, 34). These programs also involve immune and inflammatory processes and overlap with mechanisms supporting energetics, performance, and recovery in the context of physical activity and restitution (35, 36). Corrupted or untimely immunometabolic interactions are important in chronic diseases such as diabetes, multiple sclerosis, and rheumatoid arthritis (34, 37, 38). Furthermore, energy metabolism is directly linked to mechanistic elements that may contribute to fatigue, such as depletion of energy nutrients and oxygen (39, 40). Such effects compromise mitochondrial function, which has been proposed as a contributing factor in ME/CFS (41–43). We previously found indications that obstructed pyruvate flux through the central mitochondrial enzyme pyruvate dehydrogenase (PDH) plays a role in ME/CFS (15), and this is supported by other studies reporting changes in amino acid and TCA metabolites (27–29, 44). Despite these findings, we do not have a complete overview regarding changes in systemic energy metabolism in patients with ME/CFS.

The aim of the present study was to map metabolic phenotypes of ME/CFS and thereby gain insights into disease-related mechanisms. We performed comprehensive serum metabolite measurements and exploratory data analyses (EDA), and we found both common and variable alterations in the ME/CFS patient group. The abnormalities covered recognizable patterns of energy strain, as well as context-dependent signatures of deregulated metabolism. The possible clinical impact should be further investigated, as some aspects may contribute to worsening of the disease.

## Results

### Cohort characteristics

Comprehensive investigation of serum metabolites was performed employing global metabolomics and lipidomics. Differences in metabolite profiles at group and subgroup levels were further investigated using clinical data, quantitative blood biochemistry measurements, and metabolic hormone analyses. The study included 35 healthy controls (HC) and 83 patients with ME/CFS who were included in the rituximab (51 patients) or cyclophosphamide (32 patients) intervention studies (12, 45). The HC and ME/CFS groups had a similar sex and age distribution (Table 1). The mean BMIs were comparable between the groups. There was a tendency toward more individuals with BMI > 25 among the patients with ME/CFS, but there was no statistical differences in the relative distribution in BMI categories. BMI data were missing for 5 of the HC subjects. All patients with ME/CFS had been sick for more than 2 years, and the disease severity ranged from mild to severe.

### Global metabolomics

**Figure 1:**
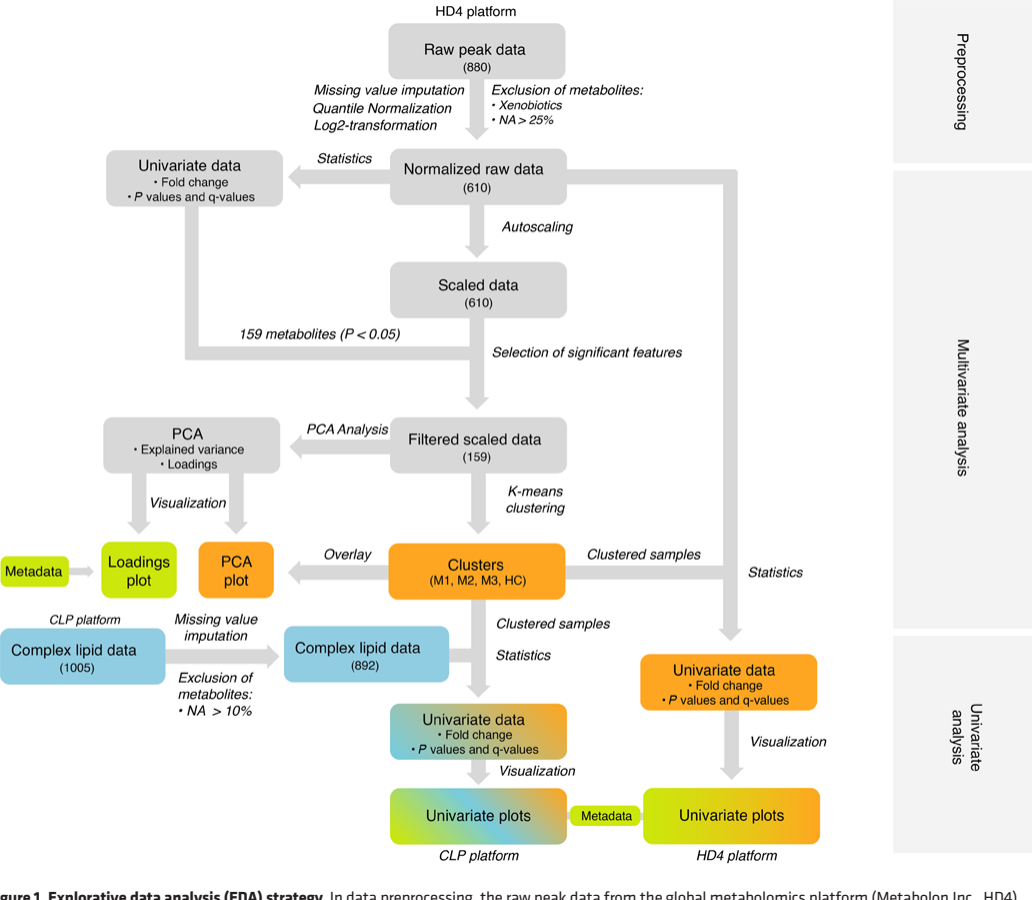
Explorative data analysis (EDA) strategy. In data preprocessing, the raw peak data from the global metabolomics platform (Metabolon Inc., HD4) was preprocessed by first excluding variables with more than 25% missing values, metabolites classified as xenobiotics, and partially characterized molecules. Missing values were then imputed for the remaining variables. The variables were subsequently quantile normalized. In multivariate analysis, initial univariate analysis was performed on the raw normalized data using 2-tailed Welch’s test revealing 159 significant metabolites (based on *P* value). Fold change was also calculated. The normalized raw data were scaled using autoscaling before further multivariate analyses were performed. The 159 significant metabolites from the first univariate analyses were used for the multivariate analyses. The k-means clustering was used generate 3 patient subsets. Principal component analysis (PCA) was performed, and a loading plot was generated. The subsets discovered in the k-means clustering was used in the visualization of the PCA plot to display the variance observed between patient subsets. Additional metadata were implemented to investigate possible impacts of factors such as such as sex and fasting state. In univariate analysis, the HD4 data set was reanalyzed to separately compare the 3 ME/CFS metabolic subsets with the HC group using the HD4 data set. These data were used to create plots for visualization. The curated complex lipid data set (Metabolon Inc., CLP) was also analyzed according to the established ME/CFS subsets.

**Figure 2:**
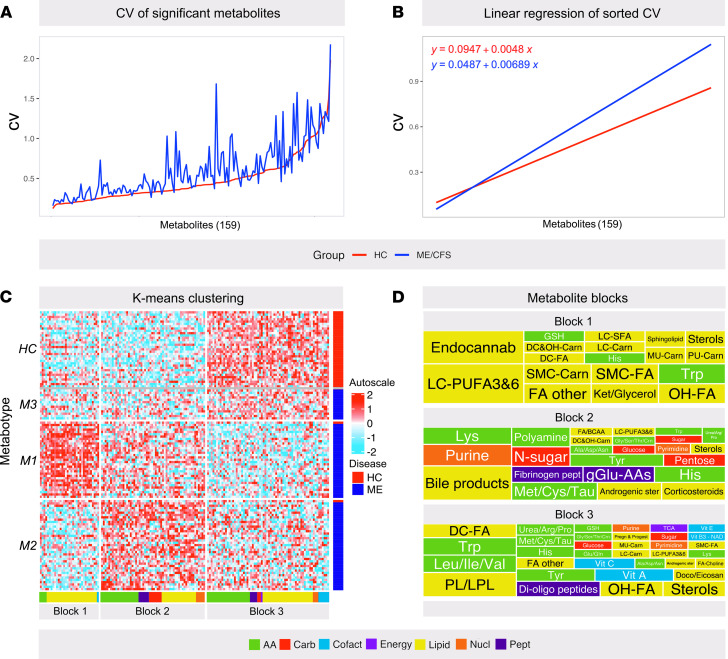
Clustering analysis of global metabolite data. Untargeted global metabolome analysis in serum from 35 healthy controls (HC) and 83 patients with ME/CFS (ME). Initial analyses identified 159 metabolites significantly different in patients with ME/CFS compared with the HC group (2-tailed Welch’s test, *P <* 0.05). (**A**) The graph illustrates the coefficient of variation (CV) for all the significantly altered metabolites. The metabolites are organized along the *x* axis according to their CV in the ME/CFS group, with increasing CV from left to right. (**B**) Linear regression of the data in **A** to assess the overall difference in data heterogeneity between the 2 groups. (**C**) Heatmap based on k-means clustering using the 159 significantly different metabolites. The heatmap shows the autoscaled levels of each metabolite in each sample, colored blue for decline and red for elevation as indicated on the vertical bar. The patients with ME/CFS clustered into 3 subsets with different metabolic phenotypes, here referred to as metabotypes (M1–M3). The M1 and M2 subsets contained the majority of patients with ME/CFS, and only 1 HC subject clustered with each of them. The third cluster contained the majority of HC subjects and the remaining patients with ME/CFS (M3; separated from HC with a gray line). The heatmap split the 159 metabolites into 3 blocks, or “signatures,” for which the involved metabolite classes are indicated (color-coded) underneath the heatmap. (**D**) “Tree map” illustrating the relative contributions (box sizes) of the metabolite classes in each metabolite block. The colors indicate the metabolite class, while the abbreviated subclasses are written inside the boxes (see Supplemental Data Set 1 for full names).

The global metabolomics analysis (HD4 platform, Metabolon Inc.) detected 880 serum compounds in the study cohort. Based on initial pilot statistical analyses, we designed a strategy for EDA aiming to unravel interesting patterns and generate hypotheses, as displayed in Figure 1. Throughout the study, statistical significance for each metabolite was evaluated based on both univariate *P* value (2-tailed Welch’s test) and multivariate adjusted *P* value (Benjamini-Hochberg adjustment; *q* value). Since this was an exploratory study, univariate *P <* 0.05 was used as the standard criterion for significance. The different data elements of the analysis are provided as listed in Supplemental Data Set 1, sheet 1 (supplemental material available online with this article; https://doi.org/10.1172/jci.insight.149217DS1), and pathway abbreviations are defined in sheet 2 of the same file. After excluding compounds with high levels of missing values, and xenobiotic molecules, the statistical analysis included 610 different compounds (Supplemental Data Set 1, sheet 3). Using univariate statistical analysis, 159 of 610 metabolites were found to be significantly different between the ME/CFS and HC groups (2-tailed Welch’s test, *P <* 0.05; 84 were *q*-significant), of which 87 of 159 had elevated and 72 of 159 had lower serum concentrations in the patients (Supplemental Data Set 1, sheet 4). The majority of affected metabolites were linked to lipid (75 of 159) and amino acid (49 of 159) metabolism. Inside both the ME/CFS and HC groups, the data heterogeneity, expressed by the coefficient of variation (CV), differed between the individual metabolites (Figure 2, A and B). Although the heterogeneity profiles in the 2 groups mirrored each other, the ME/CFS group expressed overall higher internal variation compared with the HC group, which has also been observed in other cohorts (25). Theoretically, homogenous effects in the ME/CFS group may reflect common disease mechanisms, whereas heterogeneous traits more likely express context-dependent changes that may be influenced by multiple factors.

### Multivariate analysis identified 3 different metabolic phenotypes (metabotypes) of ME/CFS

To detect possible subsets of patients with ME/CFS with different metabolic profiles, we performed k-means clustering using the 159 metabolites that had statistically significant level differences (*P <* 0.05) between the ME/CFS and HC groups to generate a heatmap (Figure 2C). The majority of the HC subjects clustered together (33 of 35 subjects), whereas 84% of the patients with ME/CFS (70 of 83 patients) clustered into 2 subgroups with clearly different metabolic phenotypes: metabotype 1 (ME-M1; 32 of 83 patients) and metabotype 2 (ME-M2; 38 of 83 patients). The remaining small group of patients with ME/CFS (metabotype 3 [ME-M3]; 13 of 83 patients) clustered together with the HC subjects. One HC subject was present in each of the ME-M1 and ME-M2 clusters. Since the ME-M1 and ME-M2 subsets represented the major and most distinct phenotypes of this cohort, they attracted primary focus throughout the study.

**Figure 3:**
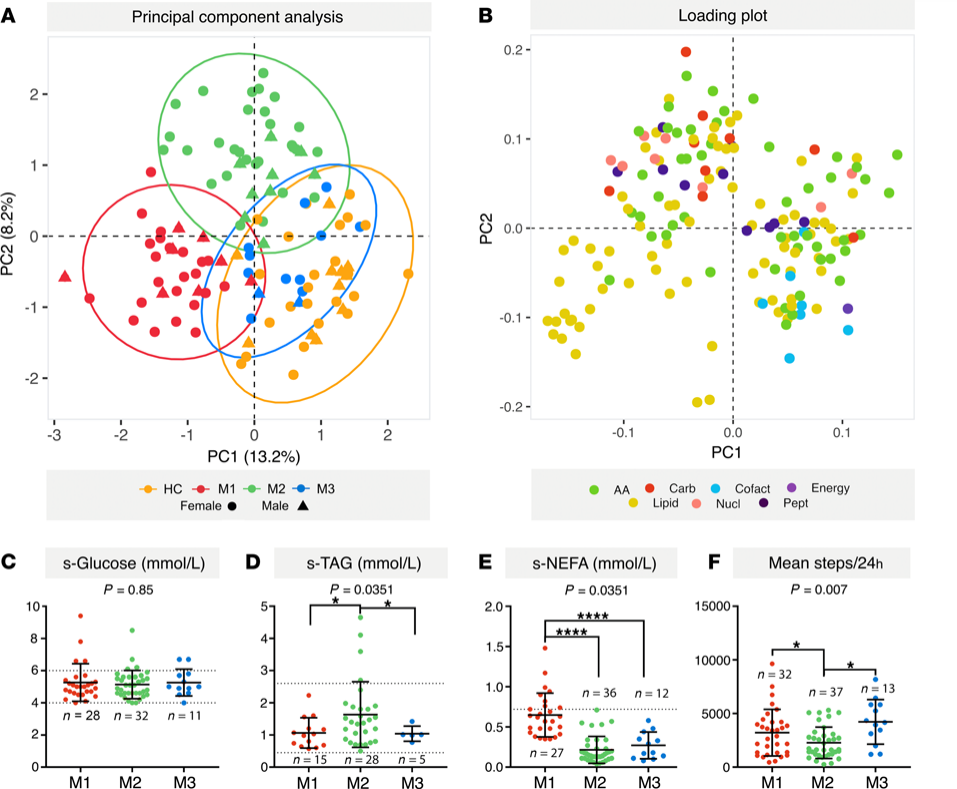
Validation of different metabotypes. Metabolomics in serum from 35 healthy controls (HC) and 83 patients with ME/CFS (ME) identified 159 significantly different metabolites (2-tailed Welch’s test, *P <* 0.05). (**A**) Principal component analysis (PCA) plot based on the significantly different metabolites. The PCA was performed independently of the introductory k-means clustering analysis. However, the clusters from the k-means analysis (in Figure 2C) have been used as an overlay to evaluate the associations and robustness of the proposed ME/CFS subsets. The sex of each subject is also indicated (dots, female; triangles, male). (**B**) Loading plot showing the contributions of the 30 main metabolites of PC1 and PC2, color-coded according to the metabolite class. (**C**–**E**) Clinical laboratory measurements of serum glucose (**C**), serum triglycerides (s-TAG) (**D**), and serum nonesterified fatty acids (s-NEFA) (**E**). Data are presented as mean ± SD, and the reference range is shown with dotted lines. (**F**) Mean steps/24 hours. *P* values; 1-way Welch’s ANOVA test, comparison of M1, M2, and M3. *P < 0.05; ****P < 0.00001; 2-tailed Welch’s test.

As an additional and independent strategy to evaluate the different metabotypes, we performed principal component analysis (PCA) using the same 159 variables described above (Figure 3A). At a group level, the patients with ME/CFS showed considerably more scattered distribution in the PCA plot compared with the HC subjects, confirming the larger data variance. There was considerable overlap between the 2 groups in the PCA plot; however, when we annotated (using colored symbols) according to the subsets from the k-means clustering, it was evident that subjects of the same metabotype grouped together. In the annotated plot, there was relatively little overlap between the HC, ME-M1, and ME-M2 clusters, whereas the ME-M3 subset positioned as a merger phenotype between the 3 others.

The 2 strategies of multivariate analysis consistently supported the division of the ME/CFS cohort into 3 subsets based on serum metabotype and simultaneously uncovered specific metabolites that were differently affected between the subsets. Based on the clustering analysis, the metabolites divided into 3 groups (referred to hereafter as metabolite blocks) with different discriminating impact on the metabotype (Supplemental Data Set 1, sheet 5). Figure 2D displays the types and relative abundance of affected metabolites in the different metabolite blocks (abbreviations provided in Supplemental Data Set 1, sheet 2). Overall, the patterns that separated the ME/CFS subsets were dominated by lipid and amino acid metabolites. This notion was further supported by the PCA. Principal component 1 (PC1) and PC2 together explained 21.4% of the variation (PC1 13.2 %, PC2 8.2%; Figure 3A) and were primarily driven by heterogeneity in lipid and amino acid metabolites (Figure 3B; the top 30 metabolites of PC1 and PC2 are provided in Supplemental Data Set 1, sheet 6). The metabolic signatures of the different metabotypes are described in more detail in the next sections.

### Evaluation of the ME/CFS metabotype subsets

We evaluated possible confounding factors that could have influenced the ME/CFS metabotypes, such as sex, BMI, medication, and critical elements of the statistical analysis. An extensive summary of these investigations is provided in Supplemental Data 1, with complementary data in Supplemental Data Set 3 (impact of missing value imputation in different subsets [group, sex, metabotype]) and Supplemental Data Set 4 (separate univariate analysis in female and male patients). The metabotypes did not associate with sex, and they were adequately expressed in both sexes. Although BMI showed some influence, the possible impact of obesity appeared weak as the metabotypes were adequately reproduced in nonobese subjects (BMI < 25). Furthermore, we did not find significant influences of age or postprandial fasting time. Based on the 185 xenobiotic molecules excluded from the multivariate clustering analysis, we found no association between metabotype and specific drugs or dietary products (Supplemental Data Set 1, sheet 11). However, as expected patients with ME/CFS generally had overall higher levels of metabolites of supportive drugs relative to the HC group; for instance, they had higher levels of nonsteroid antiinflammatory drugs (NSAIDs). To summarize, the control investigations did not reveal interactions indicating that the external factors were responsible for the multivariate clustering pattern.

Clinical laboratory data on blood biochemistry were available for most of the included patients with ME/CFS (Table 2; data according to sex in Supplemental Data 2). Although the clinical laboratory results were mainly within normal ranges, the ME/CFS metabotype subsets presented different serum lipid profiles. Blood glucose levels were similar between the ME/CFS subsets (Figure 3C). The ME-M1 subset was particularly characterized by high nonesterified fatty acid (NEFA) level and ME-M2 by high triglyceride (TAG) and low NEFA levels (Figure 3, D and E). These different blood lipid profiles support the notion that the uncovered ME/CFS metabotypes represent subclinical metabolic changes maintained by the underlying chronic pathology.

The overall mean BMI was similar between the ME/CFS and HC groups (24.4 ± 4.5 kg/m^2^ versus 23.9 ± 2.4 kg/m^2^, respectively; Table 1). The ME-M1 subset had lower mean BMI compared with the ME-M2 subset (23.1 ± 4.0 kg/m^2^ versus 25.7 ± 5.0 kg/m^2^, *P <* 0.05), but none were significantly different from the HC group. The ME-M2 group had a proportionally larger fraction of individuals with BMI > 25 compared with the HC group (55.3% versus 23.4%; Fisher’s exact test, *P <* 0.05).

Regarding severity, the cumulative proportion of patients in the 2 most severe categories (moderate/severe and severe ME/CFS) tended to be larger in the ME-M2 subset compared with the 2 other subsets, but the difference was not statistically significant (Table 1). In the ME-M3 group, the majority of patients (11 of 13) were diagnosed with mild/moderate or moderate severity, but the number of patients in this group was too low to make firm conclusions. However, there were significant differences between the subsets in the physical function scores (mean steps per 24 hours, Short form-36 Health Survey, Physical Function subscale [SF-36-PF] and self-reported function level in percent; Table 1 and Figure 3F). Based on an overall assessment, the apparent function levels were ranked in the following order: ME-M2 < ME-M1 < ME-M3. The ME-M2 patients had a mean SF-36-PF score as low as 22.2 ± 16.0.

Supported by available patient characteristics and clinical laboratory data, we concluded that the ME/CFS metabotypes discovered through global metabolomics represent 3 adequate subsets of patients with discernable and physiologically relevant differences in blood biochemistry and clinical profile.

### Metabolic characteristics of ME/CFS

**Figure 4:**
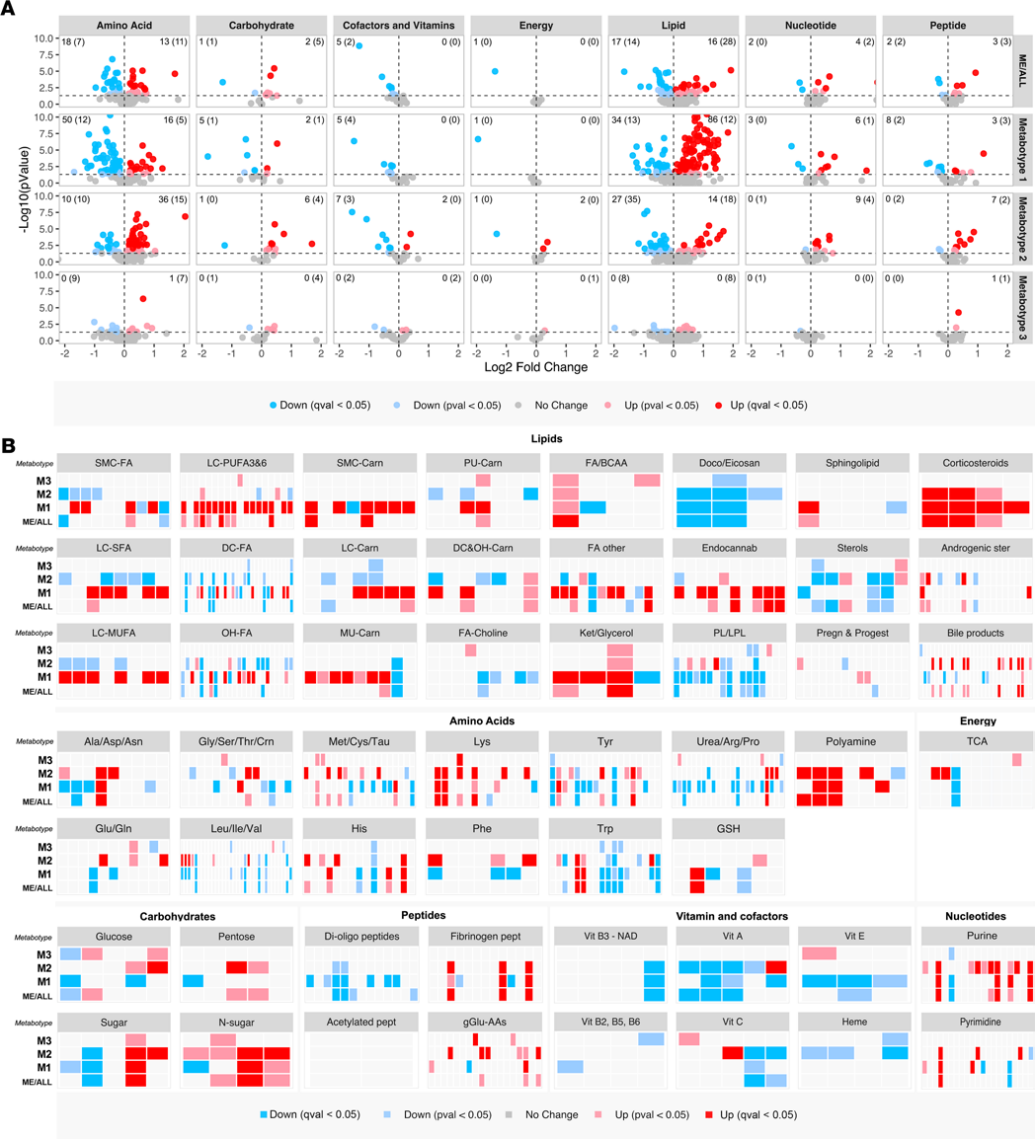
Overview of metabolite classes. The serum metabolite data set was reanalyzed to overall compare the ME/CFS group (ME/ALL) and the metabotype subsets (M1–M3) relative to the HC group. (**A**) The volcano plots give a general overview of all metabolites in a given class, with the –log_10_ of the *P* value on the *y* axis and log_2_ fold change on the *x* axis. Each dot represents a metabolite and is colored according to direction of change and significance level relative to HC as indicated (*P* < 0.05, 2-tailed Welch’s test; *q* < 0.05, adjusted *P* value). The number in each quadrant provides the respective counts of *q*-significant metabolites, and additional *P*-significant metabolites are shown in parentheses. (**B**) The tile plots display single metabolite changes according to metabolite class. Each tile represents a metabolite and is colored according to direction of change and significance level, for the entire ME/CFS group (ME/ALL) and the subsets (M1–M3) relative to HC as indicated (*P* < 0.05, 2-tailed Welch’s test; *q* < 0.05, adjusted *P* value).

**Figure 5:**
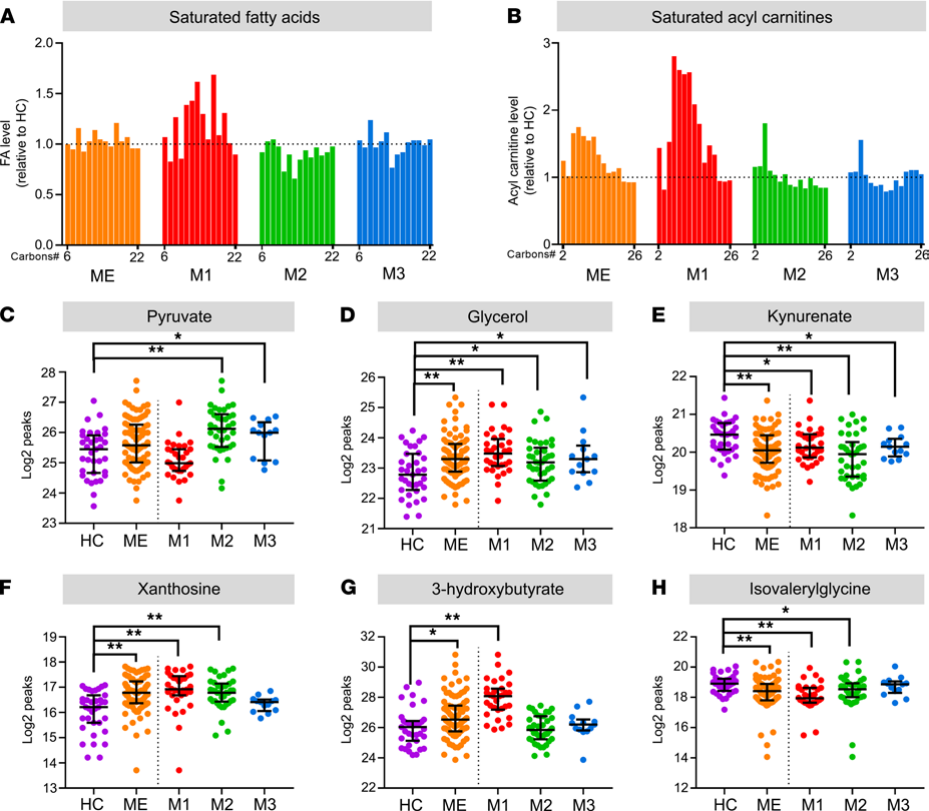
Key characteristics of ME/CFS metabotypes. Key serum molecules were investigated to evaluate contextual changes in systemic energy metabolism. (**A** and **B**) Nonesterified saturated fatty acids (**A**) and saturated acylcarnitines (**B**) in the ME/CFS group (ME) and subsets (M1-M3), calculated relative to the HC group. The fatty acid chain length is denoted on the *x* axis (Carbons#). (**C**–**H**) Serum levels of pyruvate (glucose metabolite) (**C**), glycerol (product of lipolysis) (**D**), kynurenate (Trp metabolite) (**E**), xanthosine (purine metabolite) (**F**), 3-hydroxybutyrate (β-hydroxybutyrate; ketone body) (**G**), and isovalerylglycine (BCAA product) (**H**). The metabolite levels are displayed as log_2_ transformed normalized compound peak values. Data are presented as mean ± SD. *P < 0.05, 2-tailed Welch’s test; **q <0.05, adjusted *P* value.

Since the initial establishment of the 3 ME/CFS metabotype subsets was based only on the 159 distinguishing metabolites from the univariate analyses, the entire data set was reanalyzed to explore contextual metabotype-specific changes in all the 610 metabolites (Figure 4). The metabolites were annotated to compound class as provided in Supplemental Data Set 1 (sheet 3). When compared with the HC group, the number of affected metabolites (*P <* 0.05) were 272 in the ME-M1 subset, 216 in the ME-M2 subset, and 46 in the ME-M3 subset. The higher numbers of affected metabolites in the subsets relative to the entire ME/CFS group are largely explained by the more homogenous metabolome within the subsets. Thus, the greater data variance for some metabolites in the total ME/CFS group relative to the HC group can be ascribed to differences between the metabotype subsets. The subsets showed characteristic patterns with respect to lipid and amino acid metabolites (Figure 4A). Substantial differences between the ME/CFS subsets became evident when viewing the data per metabolite subclass (Figure 4B). In Figure 5, we have extracted key parameters signaling recognizable shifts in energy metabolism, as implemented in the following sections.

In order to cluster processes that may be mechanistically or functionally connected, we evaluated common and specific features in the ME-M1 and ME-M2 subsets. A proportion of the metabolites were uniformly affected in the 2 subsets (67 of 610; Supplemental Data Set 1, sheet 7) — i.e., they were either high or low in both subsets compared the HC group. These uniform effects may theoretically be associated with a common pathomechanism, as described in further detail below. In contrast, some metabolites displayed different or even opposing effects in the ME-M1 and ME-M2 subsets; consequently, this reduced or completely neutralized the overall effects in the total ME/CFS group. Such metabotype-specific effects were particularly evident for some lipid and amino acid molecule classes (e.g., monounsaturated long-chain fatty acids), and possibly represent different context-dependent consequences of the underlying pathology.

#### Uniform metabolic effects in the ME/CFS group.

Of the 610 measured metabolites, 34 were lowered and 33 were elevated in both the ME-M1 and ME-M2 subsets relative to the HC group (Supplemental Data Set 1, sheet 7). Certain amino acid pathways were prominent among the uniformly affected metabolites. Several derivatives of BCAAs (such as isovalerylglycine) were reduced in both the ME-M1 and ME-M2 subsets (Figure 5H), and these are particularly linked to muscle energy metabolism. Specific tryptophan metabolites were either elevated or reduced in both ME/CFS subsets, possibly suggesting changes in the kynurenine pathway and NAD biosynthesis. Reduced serum kynurenate concentration was a consistent effect in all 3 subsets (Figure 5E). Also, several tyrosine metabolites were uniformly affected, including slightly elevated thyroxine level. Three metabolites of lysine were uniformly elevated in both the ME-M1 and ME-M2 subsets. These effects were accompanied by elevated levels of glycerol (Figure 5D) and short-chain acylcarnitines (butyrylcarnitine, S-3-hydroxybutyrylcarnitine), which are features known to associate with a lipolytic and ketogenic metabolic state (46). In summary, these common effects in the ME-M1 and ME-M2 subsets appear compatible with altered utilization of fatty acids and amino acids for energy fueling, as previously suggested (15), yet with contextual effects on adjacent pathways affecting the levels and transport of respective substrates and products. In agreement with a state of energy strain, there were uniform increments in several nucleotide degradation products such as adenosine and xanthosine (Figure 5F and Supplemental Data Set 1, sheet 7), which also could be consistent with alterations in amino acid metabolism (36, 47). In addition, the serum concentrations of 2 arachidonic acid derivatives, 12-hydroxyeicosatetraenoic acid (12-HETE) and 12-hydroxyheptadecatrenoic acid (12-HHTrE), were uniformly lowered in the ME/CFS subsets. There were slightly lower levels of some metabolites of vitamin A, B3, and C in both the ME-M1 and ME-M2 subsets, and there were also lower levels of metabolites of choline, which is considered an essential vitamin. Finally, some metabolites associated with corticosteroids, fibrinopeptide A and B fragments, and collagen were elevated in both subsets.

#### Metabotype ME-M1.

This subset (*n* = 32) had significantly elevated levels of 134 of 610 metabolites and lowered levels of 138 of 610 metabolites, compared with the HC group (Supplemental Data Set 1, sheet 8). A striking feature in these patients were the elevated concentrations of ketone bodies (3-hydroxybutyrate [i.e., β-hydroxybutyrate] and acetoacetate; Figure 5G), which could indicate a shift toward a more ketogenic metabolic state also involving increased fatty acid oxidation. This is coherent with increased lipolysis, as indicated by the elevated glycerol level, which was a uniform effect in the total ME/CFS group (Figure 5D). In the ME-M1 subset, there was a particularly high number of circulating lipid molecules displaying increased levels compared with the HC group (Figure 4A). These were primarily derivatives of fatty acids, with different chain length, saturation level, and adducts (Figure 4B). In agreement with clinical laboratory results, there was elevation in serum NEFAs in the ME-M1 subset (Figure 5A), and this was accompanied by increased concentration of acylcarnitines (Figure 5B). Elevated total NEFA concentration was found in both women and men in this subset (Supplemental Data 2). Lipid compounds that tended to show decreased serum levels in ME-M1 patients included metabolites of acylcholines, phospholipids and lysophospholipids, bile products, and sterols (Figure 4B). Serum glucose was slightly elevated in the ME/CFS group compared with the HC group, but it was not significantly elevated in the ME-M1 subset. The clinical laboratory results showed similar mean blood glucose concentrations in the patient subsets (Figure 3 and Table 2). Moreover, this metabotype was characterized by low levels of many amino acid metabolites compared with HC subjects (Figure 4A), including metabolites of tryptophan, the BCAAs (leucin/isoleucine/valine), arginine/proline, and phenylalanine (Figure 4B). This was also accompanied by low circulating levels of multiple di- and oligopeptides. However, affected lysine metabolites were generally increased in this subset. Minor effects were found for pyruvate (Figure 5C) and TCA cycle metabolites. In conclusion, the ME-M1 metabotype appears to reflect a lipolytic state with increased utilization of both fatty acids and amino acids as energy substrates, possibly due to ineffective carbohydrate catabolism.

#### Metabotype ME-M2.

This subset (*n* = 38) displayed significantly elevated levels of 119 of 610 metabolites and lowered levels of 97 of 610 metabolites (Supplemental Data Set 1, sheet 9). The ME-M2 patients had predominantly lower serum levels of fatty acid derivatives (Figure 4B). However, mobilization of fatty acids from stored lipids appeared to occur as the concentration of glycerol, a product of TAG lipolysis (48), was elevated, as also seen in the other 2 ME/CFS subsets (Figure 5D). In agreement with the clinical blood tests (Figure 3C), serum glucose was found normal in the ME-M2, but there was enrichment of other carbohydrates — in particular, sucrose. There was a significant increase in serum pyruvate in the ME-M2 subset (Figure 5C), possibly due to reduced mitochondrial pyruvate oxidation (15). Affected amino acid metabolites linked to pathways of alanine/aspartate/asparagine, methionine/cysteine/taurine, lysine, glutamate/glutamine, histidine, and phenylalanine, generally expressed higher levels than in the HC group (Figure 4B). For affected metabolites of tryptophan, BCAAs, glycine/serine/threonine/creatine, tyrosine and arginine/proline, there were both reduced and increased levels in this patient subset. Notably, the serum tryptophan concentration was elevated in the ME-M2 subset, in contrast to the reduced level in the ME-M1 subset, yet both expressed a decline in certain downstream intermediates, such as kynurenate (Figure 5E). Furthermore, the elevated concentration of bile products was specific for this subset, as were high levels of certain γ-glutamyl dipeptides (gGlu-AAs; Figure 4B). In conclusion, the ME-M2 metabotype shows indications of disrupted control of lipid metabolism, possibly involving compromised activity of mitochondrial oxidation pathways and consequent effects on lipid trafficking and storage.

#### Metabotype ME-M3.

This subset (*n* = 13) had relatively few features that were significantly different from the HC group. Only 21 of 610 metabolites showed reduced levels, whereas 25 of 610 metabolites showed increased levels (Figure 4; Supplemental Data Set 1, sheet 10). This was expected, since there were relatively few of these patients and they were partly clustered together with HC subjects in the multivariate analyses. The ME-M3 subset was found to reflect an intermediate state between the 2 other ME/CFS metabotypes, albeit with some more similarity with the ME-M2 phenotype.

### The lipidomes of ME/CFS

**Figure 6:**
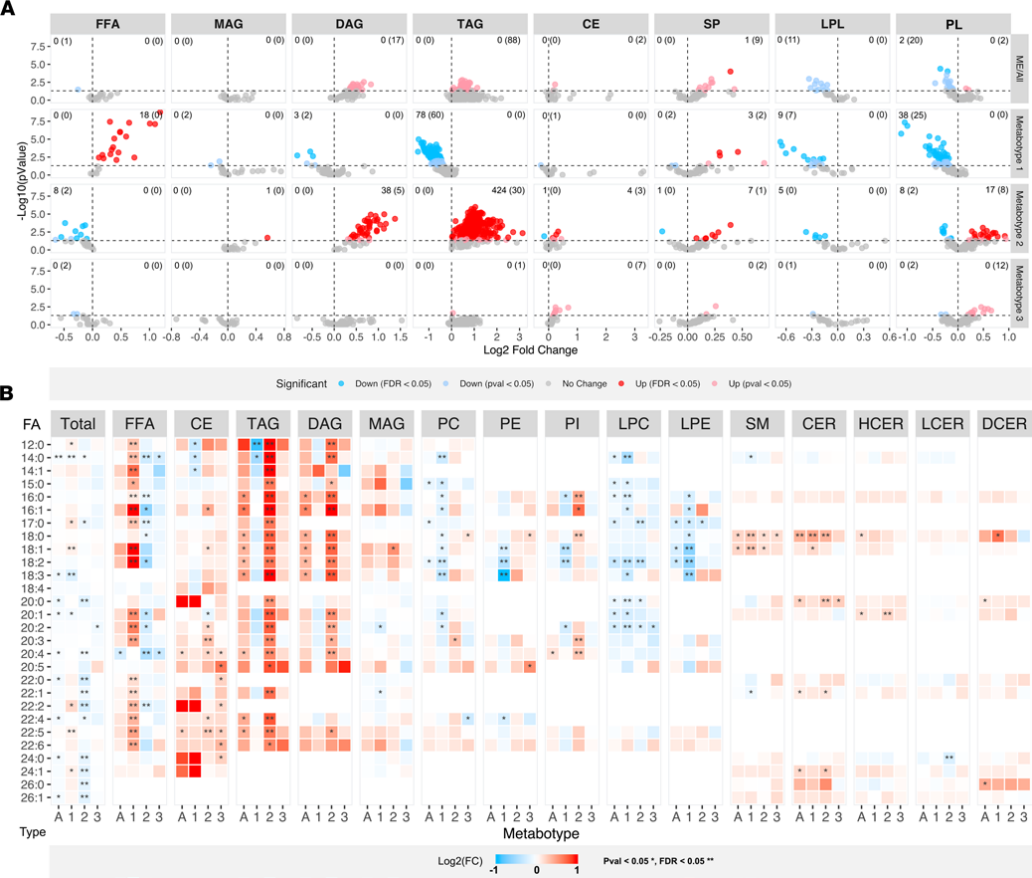
Overview of lipid classes. The serum lipidome data set was analyzed to overall compare the ME/CFS group (ME/All) and the 3 metabotype subsets (M1–M3) relative to the HC group. (**A**) The volcano plots give a general overview of all lipid molecules in a given class, with the –log_10_ of the *P* value on the *y* axis and log_2_ fold change on the *x* axis. Each dot represents a metabolite and is colored according to direction of change and significance level relative to HC as indicated (*P* < 0.05, 2-tailed Welch’s test; *q* < 0.05, adjusted *P* value). The number in each quadrant provides the respective counts of *q*-significant metabolites, and additional *p*-significant metabolites are shown in parentheses. (**B**) The relative amount of specific fatty acids (FA, first column) in the different lipid classes (column title) in the total ME/CFS group (bottom label, A) and according to metabotype subsets (M1–M3). The color of the heatmap cells display the log_2_ fold change relative to HC, as indicated. *P < 0.05, 2-tailed Welch’s test; **q < 0.05, adjusted *P* value. Total, total sum of fatty acids across all lipid classes; CE, cholesterol ester; CER, ceramide; DAG, diacylglycerol; DCER, dihydroceramide; FFA, free fatty acid (synonymous to nonesterified fatty acid, NEFA); HCER, hexosylceramide; LCER, lactosylceramide; LPC, lysophosphatidylcholine; LPE, lysophosphatidylethanolamine; LPL, lysophospholipid; MAG, monoacylglycerol; PC, phosphatidylcholine; PE, phosphatidylethanolamine; PI, phosphatidylinositol; PL, phospholipids, SM, sphingomyelin; SP, sphingolipids; TAG, triacylglycerol.

Comprehensive analysis of complex lipids was performed in serum from the same study cohort. Of the included 892 lipid species, 153 presented significantly different serum concentrations in the ME/CFS group relative to the HC group, of which 34 had lower level and 119 had higher level (Figure 6A; Supplemental Data Set 2, sheets 2 and 4). The lipid class sum concentrations indicated elevation of diacylglycerols (DAG), TAG, and dihydroceramides (DCER) and reduction of lysophosphatidylcholines (LPC) in the ME/CFS group compared with the HC group (Supplemental Data Set 2, sheet 3). However, distinct differences in lipid profile were evident when subgrouping according to the proposed ME/CFS metabotypes. Overall, there was extensive coherence between lipid-related effects observed on the global metabolomics (HD4) and lipidomics (complex lipidomics platform [CLP]) platforms. The following is a summary of the uniform and metabotype-specific changes in serum lipids in patients with ME/CFS relative to HC subjects.

#### Uniform lipidome effects in the ME/CFS group.

Only 16 of the 892 lipid species presented uniform effects in the ME-M1 and ME-M2 subsets relative to the HC group (Supplemental Data Set 2, sheet 5). Of these, 13 compounds had lower serum concentration in ME/CFS patients, and all these were phospholipid derivatives of phosphatidylcholine (PC) and phosphatidylethanolamine (PE), including LPC and lysophosphatidylethanolamine (LPE). This included several PE plasmalogen compounds, which represent a chemically and biologically unique phospholipid subclass (49). Ten of the 13 uniformly lowered phospholipid species contained at least one 18:2 acyl moiety, which represents linoleic acid, an essential fatty acid and arachidonic acid (20:4) precursor. Overall, the level of linoleic acid tended to be low in the phospholipid subclasses (PC, PE, phosphatidylinositol [PI], LPC, and LPE; Figure 6B). Only 3 single lipid species were uniformly elevated — the sphingolipids, CER(18:1), SM(18:0), and SM(18:1) — yet additional sphingolipids showed similar trends. Regarding effects on total serum fatty acid content (i.e., esterified and nonesterified), the only significant uniform change in the ME-M1 and ME-M2 subsets was reduced level of 14:0 (Figure 6B).

#### Metabotype ME-M1 lipidome.

This subset had significantly elevated serum concentration of 23 of 892 and lowered level of 227 of 892 lipid metabolites compared with the HC group (Figure 6; Supplemental Data Set 2, sheet 6). Most of the elevated metabolites were NEFAs (18 of the 25 measured free fatty acids; Figure 6A), contributing to the higher total NEFA concentration. The remaining 5 elevated compounds in the ME-M1 subset were all sphingolipids containing 18:0 or 18:1 (CER[18:0], CER[18:1], DCER[18:0], SM[18:0], SM[18:1]). The 227 lowered metabolites predominantly included species of glycerolipids (138 TAG, 5 DAG, 2 monoacylglycerols [MAG]) and phospholipids (79, including 16 lysophospholipids), in addition to SM(14:1), SM(22;1), and CE(14:1). Although the sum concentration of TAG was not significantly lowered relative to the HC group, the lowered levels of multiple single TAG are coherent with the relatively low total serum TAG concentration observed in laboratory measurements (Figure 3D). Furthermore, there was significant or trending decrease in the sum concentrations of phospholipids (PC, PI, trend for PE) and lysophospholipids (LPC, LPE) (Supplemental Data Set 2, sheet 3). There was no significant effect on total levels of sphingolipid subclasses, but there was a trend for lower total DCER level. In the NEFA fraction, there was a particular relative enrichment (mole percent) of the 16:1, 18:1, and 18:2 fatty acids, whereas the TAG fraction had significantly lower content of C12 and C14 fatty acids (Supplemental Data Set 2, sheet 10). The findings are compatible with increased mobilization and oxidation of fatty acids, as suggested by increased concentrations of free fatty acids, glycerol, and ketone bodies. The observed changes in the ME-M1 subset may mimic metabolic adaptations known to occur in response to energy strain triggered by starvation or exercise (50, 51). Theoretically, such a response could be caused by aberrant regulation of glucose catabolism, a chronic high ATP demand, or uncoupling of mitochondrial respiration leading to excessive oxidative flux and inefficient ATP production.

#### Metabotype ME-M2 lipidome.

This subset had higher concentrations of 538 of 892 lipid compounds, and lower concentrations of 27 of 892, compared with the HC group (Figure 6; Supplemental Data Set 2, sheet 7). The vast majority of the elevated compounds were different species of either TAG (454 species) or DAG (43 species; Figure 6); accordingly, there were significantly higher total serum concentrations of these 2 lipid classes. There were also higher levels of some single species — and the total class concentration — of cholesterol esters. Multiple single phospholipids (25 species of PC, PE, and PI) and sphingolipids (8 species of SM, CER, DCER, and HCER) were elevated, but the total concentrations of these compound classes were mainly unaffected, apart from the PI class presenting significant increase compared with the HC group (Supplemental Data Set 2, sheet 3). Similar to the ME-M1 subset, there was no significant effect on total levels of sphingolipid subclasses. Several single NEFAs (10 species) had lower serum concentrations in the ME-M2 subset compared with the HC group, contributing to the lower total NEFA level. Regarding phospholipids presenting lower levels, the effects were essentially coherent with the changes already described as uniform in the ME/CFS group. The elevated serum TAG concentration and lowered NEFA concentration in the ME-M2 subset compared with the HC group were confirmed in supplementary laboratory analyses (Table 2 and Figure 3D). In agreement with the higher TAG and DAG levels, there was a general increment in fatty acids esterified in these glycerolipids, across the fatty acid spectrum (Figure 6B). In summary, the metabolic phenotype of this subset was particularly characterized by a high serum concentration of TAG (women) and low NEFA (both sexes). Increased trafficking of TAG and DAG in blood is often associated with metabolic imbalance, excessive peripheral lipid accumulation, and induction of cellular and mitochondrial stress responses. Such effects have been described as consequences of various chronic diseases, also involving contexts of inflammation (34).

#### Metabotype ME-M3 lipidome.

This small subset had higher concentrations of 22 of 892 lipids, and lower concentrations of 5 of 892 lipids, compared with the HC group (Figure 6A; Supplemental Data Set 2, sheet 8). Elevation was primarily seen for certain cholesterol esters (7 species) and phospholipids (12 species). Notably, the serum level of 6 PE plasmalogens was higher in this subset, which contrasted the effects on this phospholipid subclass in the 2 other ME/CFS subsets. The few lipids presenting lower serum concentration in the ME-M3 subset compared with the HC group included 2 NEFA species (FFA[14:0], FFA[20:4]) and 3 PC derivatives (LPC[20:2], PC[16:0/22:4], PC[18:1/20:2]). This subset displayed higher total SM level, and trends of higher total cholesterol ester and PE levels, compared with the HC group. Relatively few effects were found regarding fatty acid composition of the different lipid classes (Figure 6B). It appeared that several of the affected single traits were unique to this subset compared with the others, but the possible implications are difficult to evaluate due to the low number of subjects. Viewing the overall tendencies, the ME-M3 subset appeared to be more similar to the ME-M2 subset than the ME-M1 subset, as indicated by a trending elevation of TAG.

### Hormone signatures of metabolic stress

**Figure 7:**
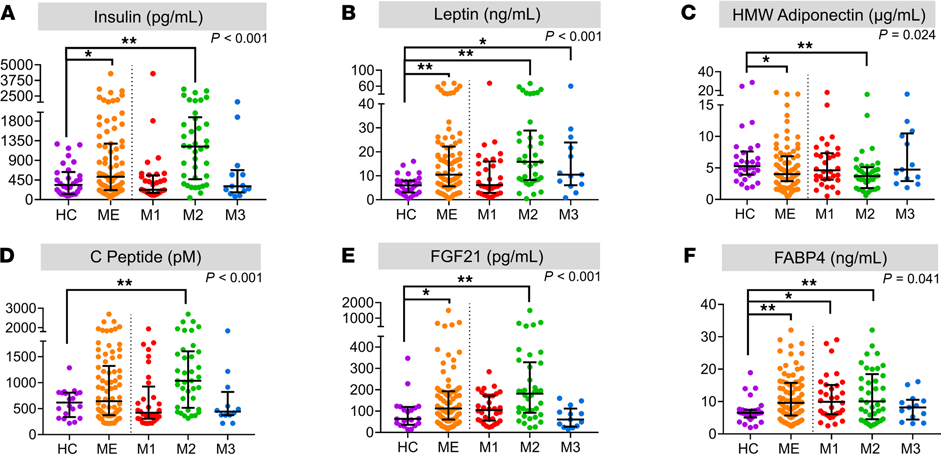
Changes in serum signaling factors linked to energy metabolism. Selected hormones potentially associated with energy strain and deregulated metabolism were measured in serum from patients with ME/CFS and HC subjects using immune-based methods. (**A**–**F**) The serum concentration was measured in 83 patients with ME/CFS and 30 HC subjects for insulin (pg/mL) (**A**), leptin (ng/mL) (**B**), HMW adiponectin (μg/mL) (**C**), C-peptide (pM) (**D**), FGF-21 (pg/mL) (**E**), and FABP4 (ng/mL) (**F**). The group median ± IQR is indicated. The shown *P* values (upper right) are for 4-group comparison of HC, M1, M2, and M3 using 1-way Kruskal-Wallis ANOVA. *P <0.05, Mann-Whitney *U* test; **q < 0.05, adjusted *P* value.

We investigated the circulating levels of selected hormones controlling energy homeostasis in contexts of physiological strain, inflammation, and pathogenesis (Figure 7). Compared with the HC group, the ME/CFS group had slightly elevated mean insulin and leptin serum levels, as well as lower high molecular weight (HMW) adiponectin (Figure 7, A–C). However, this was primarily driven by the ME-M2 subset, as no significant or trending effect was seen for the others. Accordant with increased insulin, the ME-M2 subset also had elevated mean concentration of C-peptide (Figure 7D). These specific effects on hormone levels support different regulatory contexts in the ME-M1 and ME-M2 subsets, and the findings are coherent with the observed metabolic phenotypes indicating reduced glucose utilization (ME-M1) and excessive lipid accumulation (ME-M2). Furthermore, there were increased serum concentrations of FABP4 and FGF21 in the ME/CFS group compared with the HC group (Figure 7, E and F). These factors are regarded as signals of energy strain and have been linked to metabolic disease as well as exercise (52, 53). Only the ME-M2 subset presented significant elevation of FGF21. There was a small group of patients with a particularly high level of FGF21 in the ME-M2 subset, and we suspect this may indicate an excessive burden of hepatic metabolic stress. The elevation in serum FABP4 concentration was similar in the ME-M1 and ME-M2 subsets. The observed endocrine signatures are consistent with deregulated metabolism and elevated energy strain in patients with ME/CFS, and with links to regulatory networks that may explain context-dependent responses.

## Discussion

This exploratory metabolomics study revealed a map of common and variable metabolic phenotypes of ME/CFS. The observed metabolic changes mainly fit into the paradigm of direct and indirect effects of energy strain. The physiological relevance was supported by associations with endocrine and clinical characteristics. Trough the following discussion, we suggest that energy strain may result from exertion-sensitive tissue hypoxia and leads to the systemic patterns of metabolic adaptation and compensation.

The common metabolic changes in the ME/CFS group were dominated by a relatively small number of pathways with credible impact on energy homeostasis. Elevated circulating glycerol suggests that lipolysis is induced, and this is normally observed during fasting and exercise (35, 48). In addition, several of the findings agree with altered utilization of amino acids in patients with ME/CFS, including BCAAs, tryptophan, and others. The increase in breakdown products of purine nucleotides such as adenosine and xanthosine also appeared as a possible signature of energy strain, which normally reflects increased ATP demands and altered amino acid metabolism in muscle (36). Systemic metabolic stress was supported by elevated FABP4 and FGF21, which may signal compensatory programs, as well as tissue specific responses (54, 55). Corticosteroids did not seem to be involved, since they depended on age and BMI instead of physical function scores (Supplemental Data 3) and we did not find changes in morning cortisol and ACTH in ME/CFS compared with HC. There was a particular loss of some phospholipids containing linoleic acid (18:2). This fatty acid is an essential precursor for arachidonic acid, a central messenger molecule linked to inflammation and vasodilatation. The lowering of arachidonic acid derivatives such as 12-HETE and 12-HHTrE further indicate that auto- and paracrine processes may be affected. As further discussed below, some of these effects may be associated with mechanisms likely to be involved in symptoms of ME/CFS.

The effects that were heterogeneous in the ME/CFS group expressed specific phenotypes of deregulated energy metabolism in subsets of patients. The 2 most distinct ME/CFS metabotypes, ME-M1 and ME-M2, aligned with well-known phenotypes of chronic diseases with immuno-metabolic projections (34, 37, 38). It should be kept in mind that none of the patients of our study had a clinical prediabetic or diabetic diagnosis. The ME-M1 subset presented elevated serum levels of free fatty acids (i.e., NEFA) and ketone bodies, despite normal glucose and insulin. This may resemble a context with physiological correlations to glucose starvation and exercise (33, 35). In the ME-M2 subset, a different metabolic profile was expressed by the elevated serum TAG and insulin mean levels, yet blood glucose was not affected. This may reflect low-grade signs of lipid-induced insulin resistance associated with ectopic peripheral lipid accumulation and inflammatory responses (56). Both the ME-M1 and ME-M2 subsets convincingly expressed contexts of underlying energy strain, as further supported by the elevation of metabolic stress hormones such as FGF21 and FABP4. Furthermore, we have previously suggested that impaired function of PDH may play a role in ME/CFS (15), which is regarded as a common physiological response under energy strain (57, 58). The third and minor ME/CFS subset (ME-M3) clustered together with HC, albeit with some overlap with the other 2 subsets. The 3 ME/CFS metabotypes likely reflect compatible and functionally connected contexts of compensatory adaptations that may develop in a person-specific manner. Notably, the physical function scores ranked the subsets as ME-M2 < ME-M1 < ME-M3, and there were corresponding trending differences in disease severity. Therefore, it seems possible that these metabolic contexts may influence, or be influenced by, the pattern and severity of symptoms.

Does the metabolic phenotype of ME/CFS resemble a state of elevated energetic strain? The circulating levels of amino acids may give useful information, since they are tightly linked to energy metabolism (59). A situation of energy strain, such as physical exercise, causes increased consumption of amino acids for energy purposes. Several studies, including the present, have found significant or trending reductions of energy-linked amino acids and their metabolites in blood and/or urine of patients with ME/CFS, such as seen for the BCAAs (15, 25, 27, 28, 44). Oxidation rates of BCAAs in muscle increase gradually during prolonged exercise, leading to glycogen depletion (60), which represents a well-known fatigue mechanism (39, 40, 61, 62). Amino acids also play important metabolic roles as TCA cycle anaplerotic precursors and gluconeogenetic substrates, both during exercise and recovery. The available findings in patients with ME/CFS show several similarities with recent data on the orchestrated choreography of biological processes during and after exercise (63). Such processes include the coordinated increase in rates of glycolysis, fatty acid oxidation, BCAA consumption, and adenine nucleotide catabolism during exercise in healthy individuals. The decline in circulating BCAAs continued during recovery, in contrast to the effects on glucose and fatty acids, which nearly normalized within 1 hour after the exercise. In addition to energy metabolism, multiple processes of inflammation, oxidative stress, and immune response were induced by exercise. Furthermore, the uniform elevation of purine nucleotide metabolites are of interest in this context, as AMP is an ammonia donor in muscle and normally net breakdown only occurs during conditions of working muscle such as exercise (36). These observations may suggest that metabolism is stressed in ME/CFS patients even in absence of activity. Our findings in ME/CFS ME-M1 patients showed some similarity with effects 14 hours after 3-day intensified exercise (64), including elevated free fatty acids, reduced bile products, and reduced BCAA metabolites. A chronic state of energy strain leading to persistent exercise metabolism would be expected to cause deficient capacity to accommodate additional energy demands. Such a mechanism appears relevant in ME/CFS and may theoretically contribute to key symptoms, such as fatigability and exertion intolerance.

The observed metabolic effects may be associated with tissue hypoxia caused by an underlying pathology related to an autoimmune mechanism (13). An autoantibody-mediated mechanism may influence, indirectly or directly, the fine-tuned autoregulation of blood flow required to meet the metabolic demands of tissues. Endothelial dysfunction has been shown in patients with ME/CFS (65, 66), and this was also supported in substudies linked to the CycloME and RituxME trials (67). The reduced levels of certain arachidonic acid derivatives seen in the present study may impact the vascular tone and blood flow (68). Furthermore, abnormal RBC deformability has been reported in ME/CFS, which may affect their transport through capillaries (69). These findings pointing to vascular dysfunction support that exertion-triggered tissue oxygenation may be impaired, and clearly this would contribute to lowered activity tolerance and involve mitochondrial energy metabolism. One may also speculate that symptom-generating mitochondrial effects are pathologically reinforced by exertion, as observed after excessive exercise (70). Limited, and possibly local, tissue hypoxia and lactate accumulation may also be associated with chronic inflammation (71, 72). Oxygen restriction increases the demand for TCA anaplerosis (73), which also seems to be important in ME/CFS (15). At the group level, the patients with ME/CFS in the present study displayed normal resting lactate level, suggesting that tissue hypoxia is not a dominant factor in an unprovoked context. In fact, when we grouped the patients according to metabotype, the ME-M1 subset showed slightly lower — and the ME-M2 subset slightly higher — lactate concentrations compared with the HC group. We suspect this difference in resting serum lactate between the metabotype subsets to be linked to the corresponding trends for the precursor pyruvate, rather than differences in tissue oxygenation. However, the homogenous elevation of purine nucleotide metabolites that we observed in the ME/CFS group supports that oxygen supply may be a common limiting factor (74, 75). Exercise studies have indicated that low oxygen uptake by muscle cells, and insufficient metabolic adaptations to incremental exercise, are linked to exertion intolerance in patients with ME/CFS (75–78). Reduced tissue perfusion through dysautonomia and/or inadequate autoregulation of blood flow have been found in persons with unexplained exertion intolerance, possibly via mechanisms of endothelial and microcirculatory dysfunction effects on vascular endothelium (66, 76, 77, 79, 80). In summary, we find that the metabolic changes in patients with ME/CFS are compatible with disrupted energetics enforced by tissue hypoxia on exertion. Further investigations should be performed to pursue this theory and to identify possible support strategies for improved clinical care.

Possible limitations of the study included metabolite stability and the limited accuracy of global untargeted metabolomics (81). The limitations of univariate feature selection for the purpose of clustering and identifying patient subtypes in high dimensional data sets are well known issues in the statistical community (82, 83) (Supplemental Data 1). Our comprehensive evaluation of possible cofounders such as sex, BMI, age, diet, or medication did not indicate that these were main drivers of the ME/CFS phenotypes, but they may contribute to individual variation (Supplemental Data 1). A strength of the study was that statistical analyses were strongly and independently supported by multiple layers of biochemical findings and rationale.

In summary, the study provides a map of metabolic alterations occurring in patients with ME/CFS. We find that the observed changes are compatible with elevated energy strain, for instance, caused by tissue hypoxia on exertion. The potential roles of specific pathways will have to be validated and explored in further targeted studies.

## Methods

### Patients with ME/CFS and HC.

In total, 83 patients with ME/CFS and 35 HC were included in this study. All patients fulfilled the Canadian consensus criteria for ME/CFS (1). The blood samples were collected before intervention (baseline) in 2 separate clinical trials led by Haukeland University Hospital, the “RituxME” trial (ClinicalTrials.gov NCT02229942, 2014–2017; ref. 45) and the “CycloME” trial (ClinicalTrials.gov NCT02444091, 2015–2020; ref. 12). The HC samples were collected (from 2015 to 2017) from subjects with no chronic disease or chronic medication — primarily from staff at the Department of Oncology, Haukeland University Hospital, and the Department of Biomedicine, University of Bergen. The HC group was selected to approximately match the age and sex distribution of the ME/CFS group. Biometric characteristics for HC subjects and patients with ME/CFS are summarized in Table 1, which also contains the data for subgroups of patients with ME/CFS annotated to the 3 different metabotypes developed in this study.

Blood samples were collected by venous puncture, processed according to a standardized biobank procedure as described in the trial protocols, and stored at –80°C (12, 45). All HC subjects, and 71 patients with ME/CFS, were nonfasting on sample collection. The remaining 12 ME/CFS samples were collected after overnight fasting and were included to facilitate evaluation of the possible impact of postprandial state. Clinical blood and serum analyses were performed according to standard laboratory routines at the hospital.

### Global metabolomics and lipidomics.

For the metabolite analyses, we included serum samples from all 83 patients and 35 HC subjects. The analyses were performed by Metabolon Inc. using their standard methods. The data were acquired applying 2 mass spectrometry–based analytical platforms, one global metabolite platform (HD4) providing measurement and identification of 882 compounds covering a broad spectrum of molecule and one CLP that assessed 1005 different molecular species of 14 lipid classes. The samples were analyzed in daily blocks, with several levels of sample and data quality control, such as inter-day variation correction before data normalization.

### Metabolic hormone analyses.

For metabolic hormone analyses, we included the same samples, except 5 HC, where there was an insufficient amount of serum. The serum samples were diluted, and assays were performed according to the manufacturer’s guidelines. Kits based on the Luminex multiplex bead immunoassay technology (Luminex 100 instrument, Luminex Corp.) were used to detect FABP4, insulin, HMW adiponectin, and leptin (catalog LXSAMH, R&D Systems). ELISA was used to measure FGF21 (catalog DF2100, R&D Systems) and C-peptide (catalog DICP00, R&D Systems). Measurements were performed using the Spark microplate reader (Tecan Trading AG). Data analysis was done in Excel, GraphPad Prism, and R.

### Statistics.

All data analyses were performed within the R studio environment (84), using the R programming language (85). We first removed xenobiotics and metabolites with more than 25% missing values from the data set, and the pattern of missing values was assessed by χ^2^ and Fisher exact test for group- and sex-wise comparisons. Missing values were imputed using the half-minimum method (86). The imputed data were quantile normalized, log_2_ transformed, and autoscaled. Fold changes, *P* values (2-tailed Welch’s test) and adjusted *P* values (Benjamini-Hochberg adjustment) were calculated using base functions in R. The autoscaled data were filtered based on unadjusted significant metabolites (*P* < 0.05) to reduce dimensionality in data. Row- and column-wise k-means were performed on significant features to cluster metabolites and samples, and we selected partitions of clusters guided by within-cluster sum of square (wss), gap statistic, and the silhouette method. This was visualized in a heatmap using the ComplexHeatmaps package (87), and the resulting clusters were extracted and HC organized into a separate HC cluster. These clusters were used as a visualization overlay on an independent PCA, and a loading plot was used to visualize loadings from PC1 and PC2 using ggplot2 (88). A univariate analysis was performed on the extracted clusters from the multivariate analysis. Metabolites were organized into pathways according to annotations provided by Metabolon Inc. The fold changes, *P* values, and adjusted *P* values from metabolites in each respective pathway were visualized using ggplot2. Selected biometric data were correlated to metabolites that displayed uniform changes between clusters using the Spearman method, and *P* < 0.05 was set as a cutoff. This was visualized using the ggplot package.

Data from the CLP platform were preprocessed by removing variables with more than 10% of missing values in the data set and were imputed by using the minimum observed value for each metabolite. The samples were organized into the respective clusters obtained from earlier analysis of HD4, and fold changes, *P* values (Welch’s test), and adjusted *P* values (FDR) were calculated using base functions within R. Associated metadata concerning lipid classes were obtained from Metabolon Inc. and the R package Lipidr (89). The resulting data were visualized using the ggplot2. Descriptive statistics were performed using 2-tailed Welch’s test for normally distributed data, Mann-Whitney *U* tests for variables with skewed distributions, and χ^2^ and Fisher’s exact tests for categorical variables (GraphPad Prism). Comparison of 3 or more groups was performed using 1-way ANOVA test.

### Study approval.

The clinical trials from which the biobank samples were obtained, including samples from healthy controls, were approved by the Regional Ethical Committee (Tromsø, Norway; no. 2010/1318-4, no. 2014/365, and no. 2014/1672). All patients provided written informed consent.

## Author contributions

FH, AH, ØF, OM, and KJT designed the study. ØF, OM, IGR, AF, K Sørland, KL, IH, HLT, MEG, and CS included patients in clinical studies and provided biobank samples. KR, KA, IKNP, RKB, FH, and AH performed laboratory measurements. K Sommerfelt, HPM, and OD provided scientific and technical advice. AH, FH, KJT, and ØF did the data analyses. KJT, FH, and AH wrote the paper. All authors approved the final manuscript. FH is listed first among the 2 co–first authors since he entered the project first.

## Supplementary Material

Supplemental data 1

Supplemental data 2

Supplemental data 3

Supplemental data set 1

Supplemental data set 2

Supplemental data set 3

Supplemental data set 4

## Figures and Tables

**Table 2:**
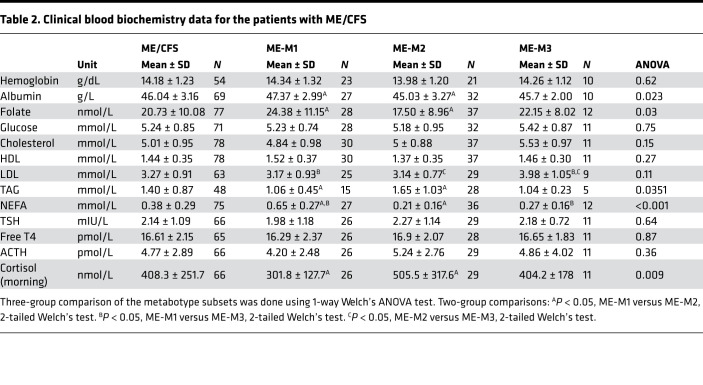
Clinical blood biochemistry data for the patients with ME/CFS

**Table 1:**
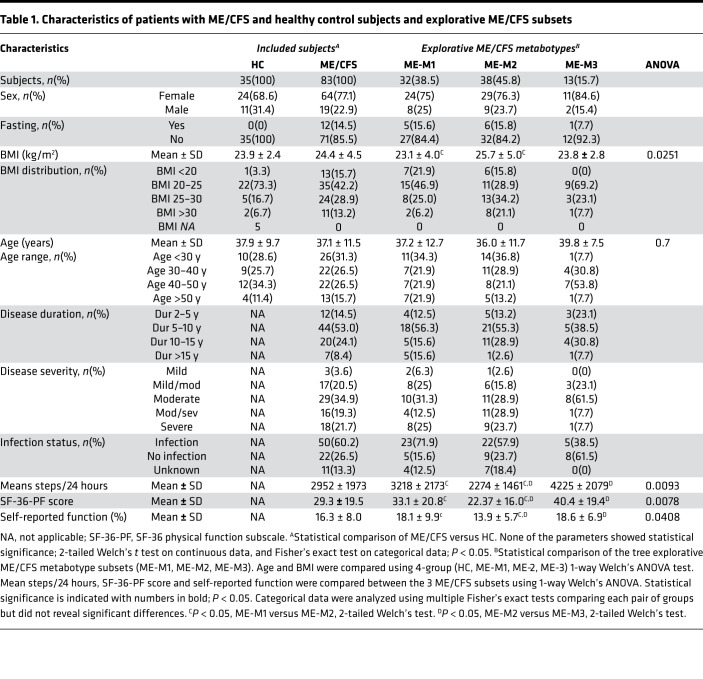
Characteristics of patients with ME/CFS and healthy control subjects and explorative ME/CFS subsets

## Version 1 08/23/2021

Electronic publication

## Version 2 08/23/2021

Added the graphical abstract.
