# Supplementary material for: A map of metabolic phenotypes in patients with myalgic encephalomyelitis/chronic fatigue syndrome
Source: JCI Insight. 2021 Aug 23;6(16):e149217. doi: 10.1172/jci.insight.149217 (PMC8409979; doi:10.1172/jci.insight.149217)
Supplement: Supplemental data 1 [file jciinsight-6-149217-s259.pdf]

Supplemental Data 1. Methodological considerations and validation of results.

Content:

1. Extended discussion of the strengths and limitations of the study
2. Possible impact of statistical method
3. Possible impact of sex
4. Possible impact of BMI
5. Possible impact of fasting, diet and drugs
6. References

1. Extended discussion of the strengths and limitations of the study:

While untargeted metabolomics can provide valuable input for hypothesis generation, care should be taken in the biological interpretation at single metabolite level, as compound detection is less accurate compared to targeted high-confidence workflows (1). For instance, we found that the amino acid effects generally trended in coherence with our previously reported targeted data in a larger ME/CFS cohort (2), but deviations were seen for several sulphur-containing amino acid metabolites. Further, factors such as variable storage time and compound stability may affect the data quality (2). The samples of the present study were collected in 2015-2017 and there may be variable metabolite decay, but control data analysis did not indicate that differences in storage time had major influence on the main findings (data not shown). The sample size is relatively small, although this is the largest broad-spectrum metabolomics study so far reported in the ME/CFS patients. The limitations of a small sample size were to some extent compensated by the large amount of data for each subject. For example, the effects on some parameters were validated with different technologies, and multifaceted cross-evaluation returned adequate support for the suggested metabotypes.

The data were preprocessed using conventional procedures, before we identified significantly different metabolites in ME/CFS patients compared to healthy subjects and clustered subjects with similar patterns of metabolite concentration shifts. We decided upon an exploratory strategy for our data analysis, and therefore applied t test statistics as filter method to select relevant features for multivariate statistics. This approach is known to be susceptible for overfitting the PCA results, so the results should be interpreted with caution. We addressed this potential bias by performing a PCA on all 610 metabolites within the dataset, which revealed that the proposed metabotypes are still highly relevant for explaining the observed variance within both sexes for the entire dataset (Section 2, below). We concluded that our EDA strategy returned statistically and biologically meaningful results, supported by patient data, lipidomics data and supplementary laboratory data. The three ME/CFS subsets with different metabotypes emerged when we focused on compounds of known identity (excluding molecules that were only partially characterized and xenobiotic substances). Although we cannot exclude some influence, the subsets were not explained by differences in age, sex, dietary supplements and medication (Sections 3-5, below). Neither did we find that fasting state influenced the ME/CFS subsets, supporting the use of non-fasting blood draws as recently recommended for metabolic profiling (3, 4).

Previous broad-spectrum metabolomics studies primarily aimed to find potential biomarkers, and therefore identified metabolites with the highest statistical probability to predict ME/CFS (5-9). Since our aim was to gain broad insight into pathways that might be involved, we used a relatively low stringency level for statistical significance when comparing single molecule concentrations in ME/CFS patient and HC subjects ($p<0.05$). Such differences in study design, as well as differences in analytical technologies and cohort size and patient characteristics, represent plausible reasons why the statistically significant metabolites differ somewhat between the various studies. In addition, the presence of different metabolic phenotypes may explain why the effects vary more for some metabolites than others within the ME/CFS group, and when comparing different cohorts and studies. For example, our findings may indicate that BMI influences context-dependent metabolic adaptations. Notably, we found that the two main ME/CFS metabotype subsets sometimes expressed opposing effects, and this neutralized the potential effect on group level for some metabolites.

2. Possible impact of statistical method

Missing imputation method: We excluded metabolites with more than 25% missing values and applied the half-minimum (HM) method for imputation. To evaluate the possible skewed impact of missing value imputation, we performed Fischers and Chi-squared analyses between different groups (ME vs HC, sex, metabotype) (Supplemental Data Set 3). This indicated that around 2 – 7% of the 610 metabolites had non-random distribution of missing values depending on the type of comparison being done. For the 159 significant variables from the univariate analyses, there was a similar percentage of metabolites displaying a non-random distribution of missing values. These results confirm that missing values imputations had negligible impact on the overall results in the multivariate analyses.

Filter method: The K-means algorithm that we applied for EDA purposes does not incorporate a feature selection method when applied outside a wrapper/embedded ML model, so a meaningful result depends on a feature selection step in advance. We used t-test statistics as filter method for our feature selection step, providing the 159 variables used for multivariate analyses. To evaluate if this cased overfitting of the results, we performed PCA on all 610 variables. The PCA on all 610 metabolites with an overlay of the proposed metabotypes (Figure 1 below) reproduced the influence of metabotypes in the total cohort, as well as in female and male separately. This supported that the choice of filter method returned adequate data, but the limitations of univariate feature selection for the purpose of clustering and identifying patient subtypes in high dimensional datasets is well known issue in the statistical community (10, 11). The variable selection approach applied in this study could potentially exclude subtle discriminating features that display a stronger joint effect with other related variables, and there are also apparent drawbacks in identifying exact number of patient subtypes with conventional clustering methods as these methods could introduce a potential bias from the investigator, and techniques to identify an exact number of clusters display between-method variation in the estimation of k.

The statistical method was supported by biologically relevant results. The different metabolic profiles found in the ME/CFS patient subsets were validated by multiple independent measurements. Further, the findings were consistent with expected effects of relevant biological contexts, based on available literature.

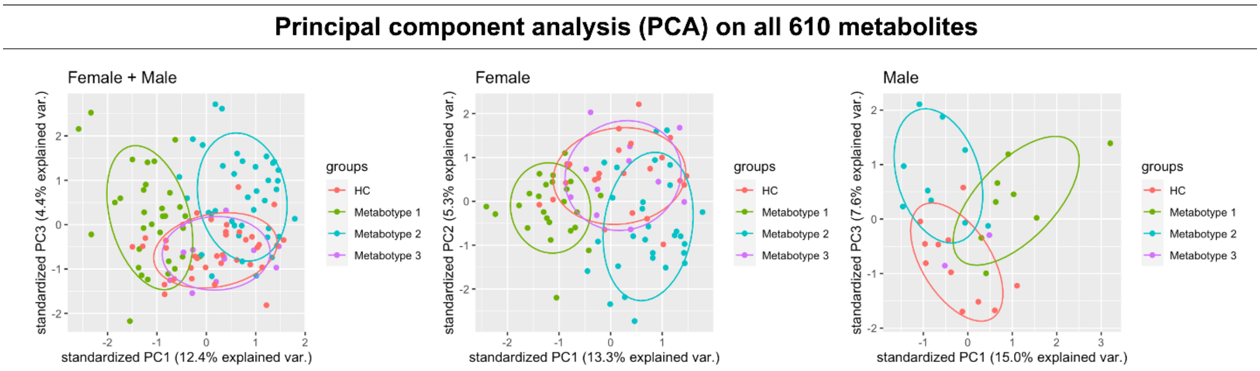

Figure 1: PCA with metaobtype overlay, based on 610 metabolites.

3. Possible impact of sex:

The possible influence of sex on the clustering results was evaluated at several levels. First, there was no significant difference in sex composition between 1) the overall HC and ME/CFS groups (Fischer's exact test; Table 1 in the article), or 2) the HC group and the ME/CFS metabotype subsets (ME-M1, ME-M2 and ME-M3) (Chi squared test and Fischer's exact test; Table 1 in the article). Next, separate multivariate analyses using the 159 significant metabolites from the original univariate analysis confirmed that the K-means clustering patterns were largely maintained in both male and female subsets compared to mixed group (Figure 2 below). Further, the metabotypes represented different subsets in both female and male in separate PCA plots, when overlaying the original metabotype annotations (Figure 2 below). We also performed separate univariate comparison of the metabolomics data (Supplemental Data Set 4) and laboratory analyses (Supplemental Data 2) in female and male. Our conclusion is that the presented ME/CFS metabotypes are not driven by sex, and are adequately expressed in both female and male.

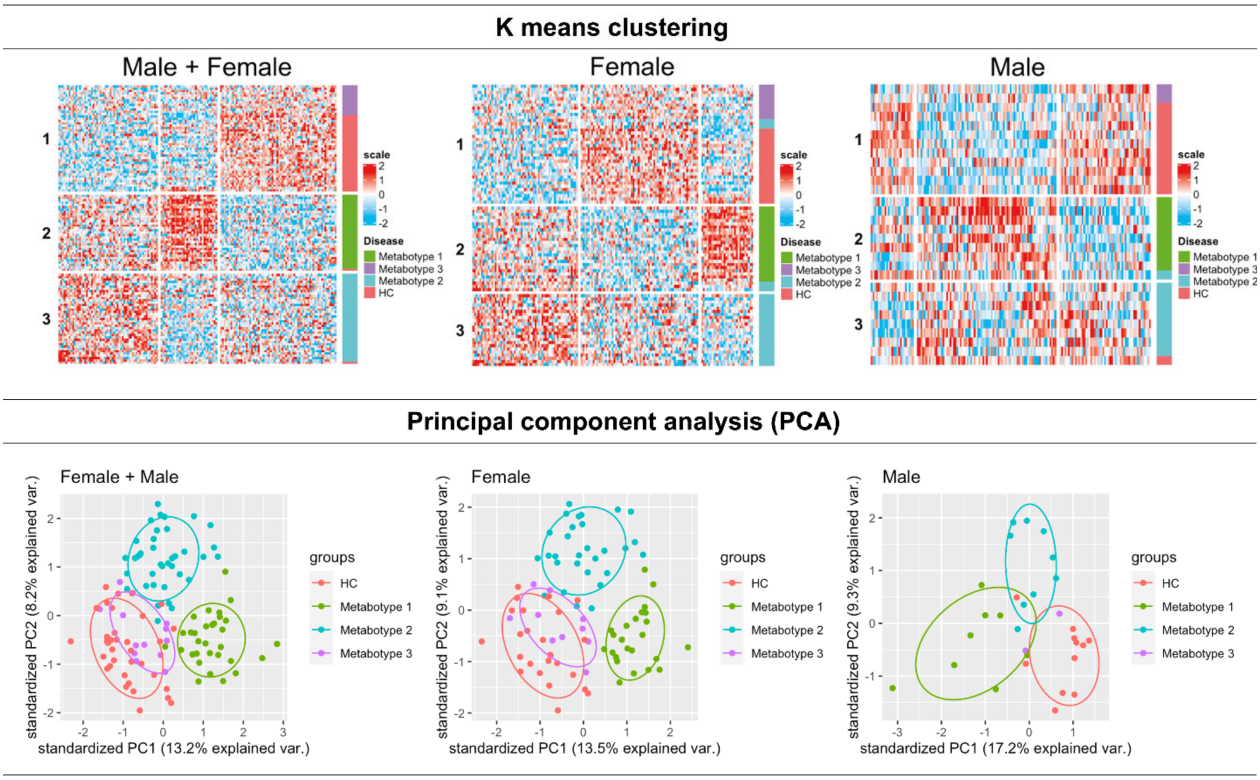

Figure 2: K-means clustering and PCA in ME/CFS and HC according to sex.

4. Possible impact of BMI

There was no significant difference in mean BMI between the HC and ME/CFS groups. However, mean BMI was significantly higher in the ME-M2 patients compared to the ME-M1 patients (25.7 vs 23.1) (Table 1 in the article). In order to perform categorical testing we divided the patients with BMI < 25 (n=48) and BMI > 25 (n=35), and found that ME-M2 had significantly higher proportion of subjects with BMI > 25 compared to HC (Fischer's exact test, $p<0.05$). To evaluate the influence of BMI on the results of the multivariate analysis, Pearson correlation analysis was performed between BMI and the principal components of the PCA (Figure 3, below). There was a significant, yet weak, association between BMI and PC2 ($r = 0.32$, $R^2 = 0.102$, $P < 0.05$). To further investigate if the results were driven by high BMI, K-means clustering and PCA were repeated after exclusion of subjects with BMI > 25 (Figure 4, below). This was performed both on group level, and separately on female and male. The results were largely consistent with the findings in the total cohort, and when dividing by sex. Hence, although we cannot exclude the possibility that there is some influence of BMI, our evaluation concludes that BMI is unlikely to be a primary determinant of the observed patterns.

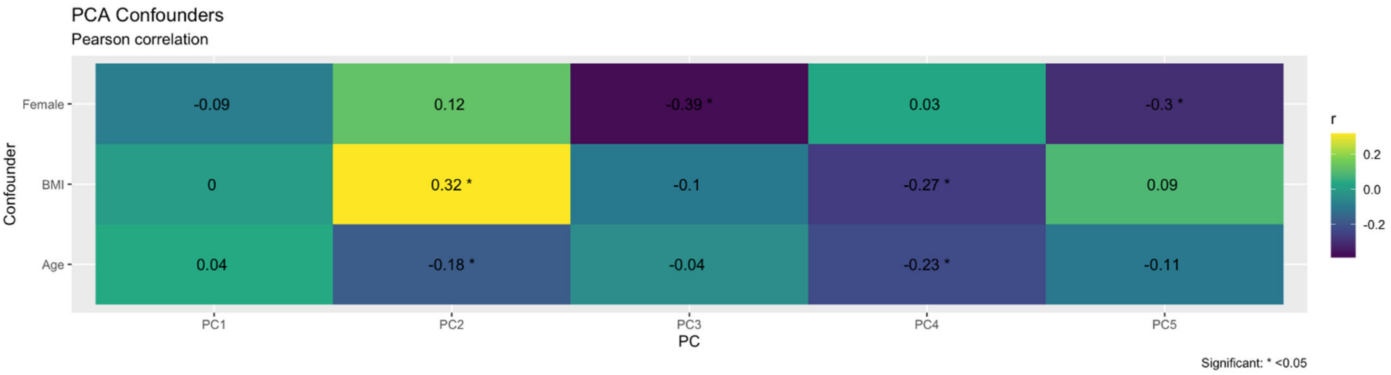

Figure 3: Pearson correlation analysis between BMI and the principal components of the PCA

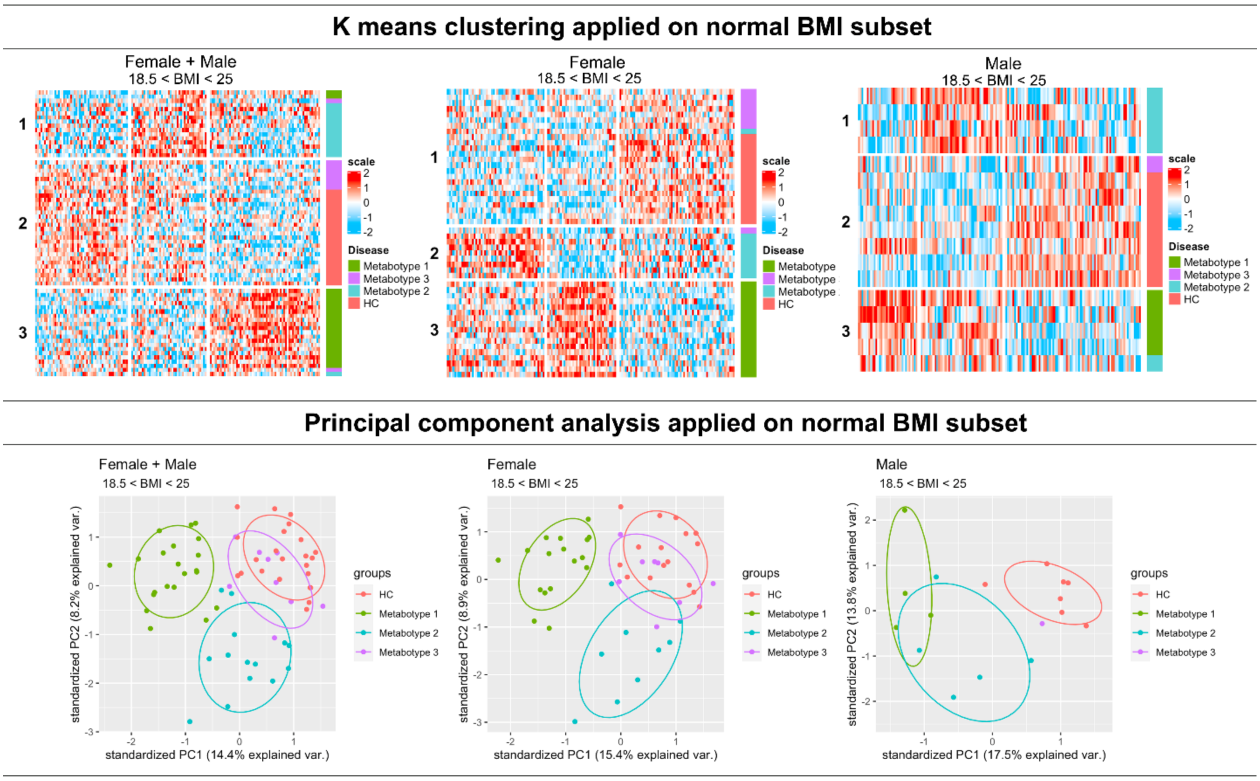

Figure 4: K-means clustering and PCA in ME/CFS and HC excluding subject with BMI>25.

5. Possible impact of fasting, diet and drugs

Fasting: The 12 patients that performed overnight fasting before sampling distributed randomly between the three subsets (Table 1 in the article). This supports that fasting state has minor influence on the proposed ME/CFS metabotypes.

Diett and drugs: To evaluate if the suggested metabotypes associated with systematic differences in diet and medication, we took advantage of the data of 185 xenobiotic molecules that were available in the global metabolomics dataset (Supplemental Data Set 1, sheet 11). To assess the use of specific drug classes, we counted the subjects having detectable levels of the associated drug derivatives. If a drug had several metabolites, we used the respective metabolite with the highest occurrence (Drug Table 1, below). The drug metabolite data was also compared with the drugs documented in the patient journal (Drug Table 2, below). As expected, the findings generally agreed with larger consumption of supportive drugs in ME/CFS patients relative to the HC group. However, the data indicated no significant differences in drug use between the ME/CFS metabotype subsets regarding analgesics, gastro-esophagal-reflux drugs, allergy medication and anti-epileptics. Yet, it appears that our strategy based on drug metabolites may have overestimated the number of users of certain drugs. For instance, metoprolol (or its metabolites) was detected in 22.9% of the patients and 8.6% of the HC subjects, whereas the number of patients that had metoprolol documented in their medical files was only 3.6%. Further, paracetamol derivatives were detected in 84.4% and 84.2% for ME-m1 and ME-m2 respectively, and in 97.1% of the HC group. For all other analgesics there were proportionally fewer observations in the HC group. Zolpidem (a sedative/hypnotic drug used for anxiety/sleep) was detected in a larger proportion of ME-m2 patient (15.8%) compared to ME-m1 (3.1%), but this was not statistically significant due to few observations.

Evaluation of xenobiotic compounds originating from foods revealed lower levels of metabolites related to peppers (such as piperin and 2-piperidinone) and caffeine in ME/CFS patients compared to HC subjects (Figure 4, below). There were also lower levels of a metabolite related to consumption of cereal and milk products (methyl glucopyranoside) in the patients. The general tendency of low dietary xenobiotics levels may agree with a low total dietary load, as would agree with limited caloric expenditure due to the debilitating condition. Of importance for the present study, the dietary xenobiotics patterns were similar among the ME-M1 and ME-M2 subsets, minimizing the probability that a systematic difference in xenobiotics causes the observed metabolic phenotypes.

Drug Table 1: Drugs detected by the HD4 platform. Compounds identified as drugs or drug metabolites by the global metabolomics platform where used to determined the number of patients or controls positive for each drug detected in the dataset. For each drug, either the drug itself or the drug metabolite with the highest number of positive observations were counted.

	HC		ME/CFS		m1		m2		m3	
	N	%	N	%	N	%	N	%	N	%
**GASTRIC**										
**RANITIDINE**	1	2.86	1	1.20	1	3.13	0	0.00	0	0.00
**OMEPRAZOLE**	0	0.00	3	3.61	1	3.13	2	5.26	0	0.00
**PANTOPRAZOLE**	1	2.86	2	2.41	1	3.13	1	2.63	0	0.00
**LANSOPRAZOLE**	1	2.86	3	3.61	1	3.13	2	5.26	0	0.00
**ANALGESICS**										
**IBUPROFEN**	3	8.57	22	26.51	8	25.00	10	26.32	4	30.77
**NAPROXEN**	1	2.86	6	7.23	3	9.38	2	5.26	1	7.69
**TRAMADOL**	1	2.86	11	13.25	5	15.63	6	15.79	0	0.00
**PARACETAMOL**	34	97.14	71	85.54	27	84.38	32	84.21	12	92.31
**ASTHMA/ALLERGY**										
**SALMETEROL**	1	2.86	1	1.20	1	3.13	0	0.00	0	0.00
**CETIRIZINE**	0	0.00	7	8.43	3	9.38	3	7.89	1	7.69
**CARDIOVASCULAR**										
**METOPROLOL**	3	8.57	19	22.89	9	28.13	7	18.42	3	23.08
**ANTIEPILEPTICS**										
**LAMOTRIGINE**	0	0.00	2	2.41	1	3.13	1	2.63	0	0.00
**GABAPENTIN**	0	0.00	2	2.41	1	3.13	1	2.63	0	0.00
**SEDATIVES**										
**ZOLPIDEM**	0	0.00	7	8.43	1	3.13	6	15.79	0	0.00
**ANTIDEPRESSANTS**										
**MAPROTILINE**	0	0.00	5	6.02	3	9.38	1	2.63	1	7.69
**SERTRALINE**	0	0.00	2	2.41	0	0.00	1	2.63	1	7.69
**MIRTAZAPINE**	0	0.00	1	1.20	0	0.00	0	0.00	1	7.69
**STIMULANTS**										
**METHYLPHENIDATE**	0	0.00	1	1.20	0	0.00	1	2.63	0	0.00
THESE DRUG METABOLITES SHOWED SIGNIFICANT DISCREPANCY WITH DRUG PRESCRIPTIONS AND PRESUMABLY HAVE OTHER ORIGIN:										
WARFARIN	29	82.86	62	74.70	22	68.75	29	76.32	11	84.62
CARBOCISTEINE	35	100.00	76	91.57	30	93.75	35	92.11	11	84.62
HYDROQUINONE	35	100.00	83	100.00	32	100.00	38	100.00	13	100.00

Drug Table 2: Drugs documented in the patient Journal. The drugs documented in patient journals were identified by their ATC codes and counted.

		ME/CFS		m1		m2		m3	
**DRUG**	**ATC**	**N(74)**	**%**	**N(28)**	**%**	**N(38)**	**%**	**N(8)**	**%**
**DIGESTIVE TRACT**									
**GASTRO-ESOPHAGAL-REFLUX DISEASE (TOTAL)**	A02B	11	14.86	6	21.43	4	10.53	1	12.50
**RANITIDINE**	A02BA02	1	1.35	1	3.57	0	0.00	0	0.00
**PANTOPRAZOLE**	A02BC02	4	5.41	2	7.14	2	5.26	0	0.00
**LANSOPRAZOLE**	A02BC03	1	1.35	0	0.00	0	0.00	1	12.50
**ESOMEPRAZOLE**	A02BC05	5	6.76	3	10.71	2	5.26	0	0.00
**ANTIEMETIC/NAUSEA**	A03F/A04A								
**METOCLOPRAMIDE**	A03FA01	2	2.70	1	3.57	1	2.63	0	0.00
**ONDANSETRON**	A04AA01	1	1.35	1	3.57	0	0.00	0	0.00
**CONSTIPATION**	A06A								
**LACTULOSE**	A06AD11	1	1.35	0	0.00	1	2.63	0	0.00
**MACROGOL, COMBINATIONS**	A06AD65	1	1.35	1	3.57	0	0.00	0	0.00
**INTESTINAL ANTIINFLAMMATORY**	A07E								
**MESALAZINE**	A07EC02	2	2.70	2	7.14	0	0.00	0	0.00
**BALSALAZIDE**	A07EC04	1	1.35	1	3.57	0	0.00	0	0.00
**SUPPLEMENTS**									
**MULTIVITAMINS**	A11BA	13	17.57	4	14.29	8	21.05	1	12.50
**VITAMIN D AND A + COMBINATIONS**	A11CC	11	14.86	5	17.86	6	15.79	0	0.00
**VITAMIN B1 + INCLUDING B6 AND B12**	A11D	2	2.70	0	0.00	2	5.26	0	0.00
**VITAMIN B-COMPLEX + INCL. COMBINATIONS**	A11EA	3	4.05	2	7.14	1	2.63	0	0.00
**VITAMIN C**	A11GA	5	6.76	2	7.14	3	7.89	0	0.00
									0.00
**VITAMIN B12 AND FOLIC ACID (USPECIFIED)**	B03B	2	2.70	1	3.57	1	2.63	0	0.00
**CYANOCOBALAMIN**	B03BA01	2	2.70	1	3.57	1	2.63	0	0.00
**HYDROXOCOBALAMIN**	B03BA03	5	6.76	2	7.14	3	7.89	0	0.00
**FOLIC ACID**	B03BB01	1	1.35	0	0.00	1	2.63	0	0.00
**CALCIUM**	A12AX	3	4.05	1	3.57	2	5.26	0	0.00
**POTASSIUM**	A12B	1	1.35	1	3.57	0	0.00	0	0.00
**IRON PREPARATIONS**	B03A	4	5.41	1	3.57	2	5.26	1	12.50
**MAGNESIUM (DIFFERENT SALTS IN COMBINATION)**	A12CC30	8	10.81	2	7.14	5	13.16	1	12.50
**OTHER MINERAL SUPPLEMENTS**	A12C	4	5.41	1	3.57	3	7.89	0	0.00
**HORMONAL THERAPIES**									

**HORMONAL CONTRACEPTIVES FOR SYSTEMIC USE (TOTAL)**	G03A	17	22.97	6	21.43	9	23.68	2	25.00
**UNSPECIFIED**	G03A	1	1.35	1	3.57	0	0.00	0	0.00
**LEVONORGESTREL AND ETHINYLESTRADIOL**	G03AA07	3	4.05	0	0.00	2	5.26	1	12.50
**DESOGESTREL AND ETHINYLESTRADIOL**	G03AA09	4	5.41	2	7.14	2	5.26	0	0.00
**DROSPIRENONE AND ETHINYLESTRADIOL**	G03AA12	4	5.41	0	0.00	4	10.53	0	0.00
**MEDROXYPROGESTERONE**	G03AC06	1	1.35	1	3.57	0	0.00	0	0.00
**ETONOGESTREL**	G03AC08	1	1.35	1	3.57	0	0.00	0	0.00
**DESOGESTREL**	G03AC09	3	4.05	1	3.57	1	2.63	1	12.50
**LOCAL**									
**PLASTIC IUD WITH PROGESTERONE**	G02BA03	1	1.35	1	3.57	0	0.00	0	0.00
**OTHER SYSTEMIC FEMALE SEX HORMONE (TOTAL)**	G03	5	6.76	3	10.71	2	5.26	0	0.00
**ESTRADIOL**	G03CA03	1	1.35	1	3.57	0	0.00	0	0.00
**ESTRIOL**	G03CA04	1	1.35	1	3.57	0	0.00	0	0.00
**NORETHISTERONE AND ESTROGEN**	G03FA01	1	1.35	1	3.57	0	0.00	0	0.00
**CYPROTERONE AND ESTROGEN**	G03HB01	2	2.70	0	0.00	2	5.26	0	0.00
**THYROID PREPARATIONS (TOTAL)**	H03A	5	6.76	2	7.14	3	7.89	0	0.00
**LEVOTHYROXINE SODIUM**	H03AA01	3	4.05	2	7.14	1	2.63	0	0.00
**LIOTHYRONINE SODIUM**	H03AA02	1	1.35	0	0.00	1	2.63	0	0.00
**THYROID GLAND PREPARATIONS**	H03AA05	1	1.35	0	0.00	1	2.63	0	0.00
**ANALGESICS AND ANTIPYRETICS**									
**NSAIDs SYSTEMIC**	M01A								
**DICLOFENAC**	M01AB05	1	1.35	0	0.00	1	2.63	0	0.00
**DICLOFENAC, COMBINATIONS**	M01AB55	1	1.35	0	0.00	1	2.63	0	0.00
**PIROXICAM**	M01AC01	1	1.35	0	0.00	1	2.63	0	0.00
**IBUPROFEN**	M01AE01	25	33.78	8	28.57	12	31.58	5	62.50
**NAPROXEN**	M01AE02	3	4.05	1	3.57	2	5.26	0	0.00
**KETOPROFEN**	M01AE03	1	1.35	0	0.00	1	2.63	0	0.00
**NAPROXEN AND ESOMEPRAZOLE**	M01AE52	5	6.76	2	7.14	3	7.89	0	0.00
**NSAIDs TOPICAL**	M02A								
**KETOPROFEN**	M02AA10	1	1.35	0	0.00	1	2.63	0	0.00
**DICLOFENAC**	M02AA15	2	2.70	1	3.57	1	2.63	0	0.00
**OPIOIDS**	N02A								
**BUPRENORPHINE**	N02AE01	1	1.35	0	0.00	1	2.63	0	0.00
**CODEIN (IN COMBINATION WITH PARACETAMOL)**	N02AJ06	12	16.22	6	21.43	6	15.79	0	0.00
**TRAMADOL**	N02AX02	8	10.81	4	14.29	4	10.53	0	0.00
**OTHER ANALEGESICS AND ANTIPYRETICS**	N02B								
**ACETYLSALICYLIC ACID**	N02BA01	1	1.35	1	3.57	0	0.00	0	0.00
**PHENAZONE, COMBINATIONS EXCL. PSYCHOLEPTICS**	N02BB51	1	1.35	1	3.57	0	0.00	0	0.00
**PARACETAMOL**	N02BE01	29	39.19	11	39.29	15	39.47	3	37.50

**ANTIMIGRAINE**	N02C								
**SUMATRIPTAN**	N02CC01	7	9.46	1	3.57	5	13.16	1	12.50
**ZOLMITRIPTAN**	N02CC03	1	1.35	0	0.00	0	0.00	1	12.50
**RIZATRIPTAN**	N02CC04	4	5.41	1	3.57	2	5.26	1	12.50
**ELETRIPTAN**	N02CC06	1	1.35	0	0.00	0	0.00	1	12.50
**CLONIDINE**	N02CX02	3	4.05	1	3.57	2	5.26	0	0.00
**CNS SUPPRESSIVE/STIMULATING DRUGS**									
**ANTIEPILEPTICS**	N03A								
**LAMOTRIGINE**	N03AX09	1	1.35	0	0.00	1	2.63	0	0.00
**GABAPENTIN**	N03AX12	2	2.70	1	3.57	1	2.63	0	0.00
**ANTIPSYCHOTICS/ANXIOLYTICS/HYPNOTICS/SEDATIVE**	N05A/B/C								
**QUETIAPINE**	N05AH04	1	1.35	1	3.57	0	0.00	0	0.00
**DIAZEPAM**	N05BA01	1	1.35	1	3.57	0	0.00	0	0.00
**OXAZEPAM**	N05BA04	3	4.05	0	0.00	3	7.89	0	0.00
**HYDROXYZINE**	N05BB01	1	1.35	0	0.00	1	2.63	0	0.00
**NITRAZEPAM**	N05CD02	2	2.70	2	7.14	0	0.00	0	0.00
**FLUNITRAZEPAM**	N05CD03	1	1.35	1	3.57	0	0.00	0	0.00
**ZOPICLONE**	N05CF01	17	22.97	7	25.00	7	18.42	3	37.50
**ZOLPIDEM**	N05CF02	7	9.46	1	3.57	6	15.79	0	0.00
**MELATONIN**	N05CH01	17	22.97	13	46.43	3	7.89	1	12.50
**ANTIDEPRESSANTS (TOTAL)**	N06A	12	16.22	5	17.86	6	15.79	1	12.50
**TRIMIPRAMINE**	N06AA06	1	1.35	0	0.00	1	2.63	0	0.00
**AMITRIPTYLINE**	N06AA09	3	4.05	2	7.14	1	2.63	0	0.00
**SERTRALINE**	N06AB06	1	1.35	0	0.00	1	2.63	0	0.00
**ESCITALOPRAM**	N06AB10	3	4.05	1	3.57	2	5.26	0	0.00
**OXITRIPTAN**	N06AX01	1	1.35	1	3.57	0	0.00	0	0.00
**MIANSERIN**	N06AX03	3	4.05	1	3.57	1	2.63	1	12.50
**OTHER**									
**METHYLPEHNIDATE**	N06BA04	1	1.35	0	0.00	1	2.63	0	0.00
**NALTREXONE**	N07BB04	4	5.41	2	7.14	1	2.63	1	12.50
**PRAMIPEXOLE**	N04BC05	1	1.35	0	0.00	0	0.00	1	12.50
**ALLERGY/ASTHMA**									
**DECONGESTANT NASAL PREPARATIONS FOR TOPICAL USE**	R01A								
**XYLOMETAZOLINE**	R01AA07	1	1.35	1	3.57	0	0.00	0	0.00
**LEVOCABASTINE**	R01AC02	1	1.35	0	0.00	1	2.63	0	0.00
**FLUTICASONE**	R01AD08	2	2.70	0	0.00	1	2.63	1	12.50
**MOMETASONE**	R01AD09	7	9.46	2	7.14	4	10.53	1	12.50
**TRIAMCINOLONE**	R01AD11	1	1.35	1	3.57	0	0.00	0	0.00
**FLUTICASONE FUROATE**	R01AD12	3	4.05	2	7.14	1	2.63	0	0.00

**PHENYLPROPANOLAMINE (NASAL DEGONCESTANT, SYSTEMIC)**	R01BA01	2	2.70	0	0.00	2	5.26	0	0.00
**INHALANTS FOR FOR OBSTRUCTIVE AIRWAY DISEASE**	R03A/B								
**SALBUTAMOL**	R03AC02	6	8.11	2	7.14	3	7.89	1	12.50
**SALMETEROL AND FLUTICASONE**	R03AK06	2	2.70	2	7.14	0	0.00	0	0.00
**FORMOTEROL AND BUDESONIDE**	R03AK07	2	2.70	1	3.57	1	2.63	0	0.00
**CICLESONIDE**	R03BA08	1	1.35	0	0.00	1	2.63	0	0.00
**DRUGS FOR SYSTEMIC USE, OBSTRUCTIVE AIRWAY DISEASE**	R03C/D								
**TERBUTALINE**	R03CC03	1	1.35	1	3.57	1	2.63	0	0.00
**THEOPHYLLINE**	R03DA04	1	1.35	0	0.00	1	2.63	0	0.00
**MONTELUKAST**	R03DC03	1	1.35	0	0.00	1	2.63	0	0.00
**ANTI-COUGH**									
**ETHYLMORPHINE**	R05DA01	1	1.35	0	0.00	1	2.63	0	0.00
**ANTIHISTAMINES FOR SYSTEMIC USE**	R06A								
**DEXCHLORPHENIRAMINE**	R06AB02	1	1.35	0	0.00	1	2.63	0	0.00
**ALIMEMAZINE**	R06AD01	5	6.76	4	14.29	1	2.63	0	0.00
**PROMETHAZINE**	R06AD02	1	1.35	0	0.00	1	2.63	0	0.00
**CETIRIZINE**	R06AE07	7	9.46	4	14.29	3	7.89	0	0.00
**LORATADINE**	R06AX13	2	2.70	0	0.00	1	2.63	1	12.50
**DESLORATADINE**	R06AX27	8	10.81	4	14.29	3	7.89	1	12.50
**DECONGESTANTS AND ANTIALLERGICS**	S01G								
**TETRYZOLINE, COMBINATIONS**	S01GA52	1	1.35	0	0.00	1	2.63	0	0.00
**CROMOGLICIC ACID**	S01GX01	1	1.35	0	0.00	1	2.63	0	0.00
**LEVOCABASTINE**	S01GX02	3	4.05	0	0.00	3	7.89	0	0.00
**KETOTIFEN**	S01GX08	1	1.35	0	0.00	0	0.00	1	12.50
**OTHER**									
**DIFFERENT CORTICOSTEROID PREPARATIONS**									
**TRIAMCINOLONE (SYSTEMIC CORTICOSTEROID)**	H02AB08	1	1.35	1	3.57	0	0.00	0	0.00
**TOPICAL CORTICOSTEROIDS (BETAMETHASONE)**	D07A	3	4.05	1	3.57	1	2.63	1	12.50
**TOPICAL CORTICOSTEROIDS (PREDNISOLONE)**	C05AA04	1	1.35	0	0.00	0	0.00	1	12.50
**CARDIAC DISEASE/HYPERTENSION**	C0								
**GLYCERYL TRINITRATE**	C01DA02	1	1.35	1	3.57	0	0.00	0	0.00
**FUROSEMIDE**	C03CA01	2	2.70	1	3.57	1	2.63	0	0.00
**METOPROLOL**	C07AB02	3	4.05	3	10.71	0	0.00	0	0.00
**LERCANIDIPINE**	C08CA13	1	1.35	1	3.57	0	0.00	0	0.00
**ATORVASTATIN**	C10AA05	1	1.35	1	3.57	0	0.00	0	0.00
**EZETIMIBE**	C10AX09	1	1.35	1	3.57	0	0.00	0	0.00
**ANTIBIOTICS/ANTIINFECTIVES**									
**MECILLINAM**	J01CA11	1	1.35	0	0.00	1	2.63	0	0.00
**METHENAMINE**	J01XX05	1	1.35	0	0.00	1	2.63	0	0.00

**CHLORAMPHENICOL**	S01AA01	2	2.70	0	0.00	2	5.26	0	0.00
**ANTIGOUT**									
**ALLOPURINOL**	M04AA01	1	1.35	1	3.57	0	0.00	0	0.00

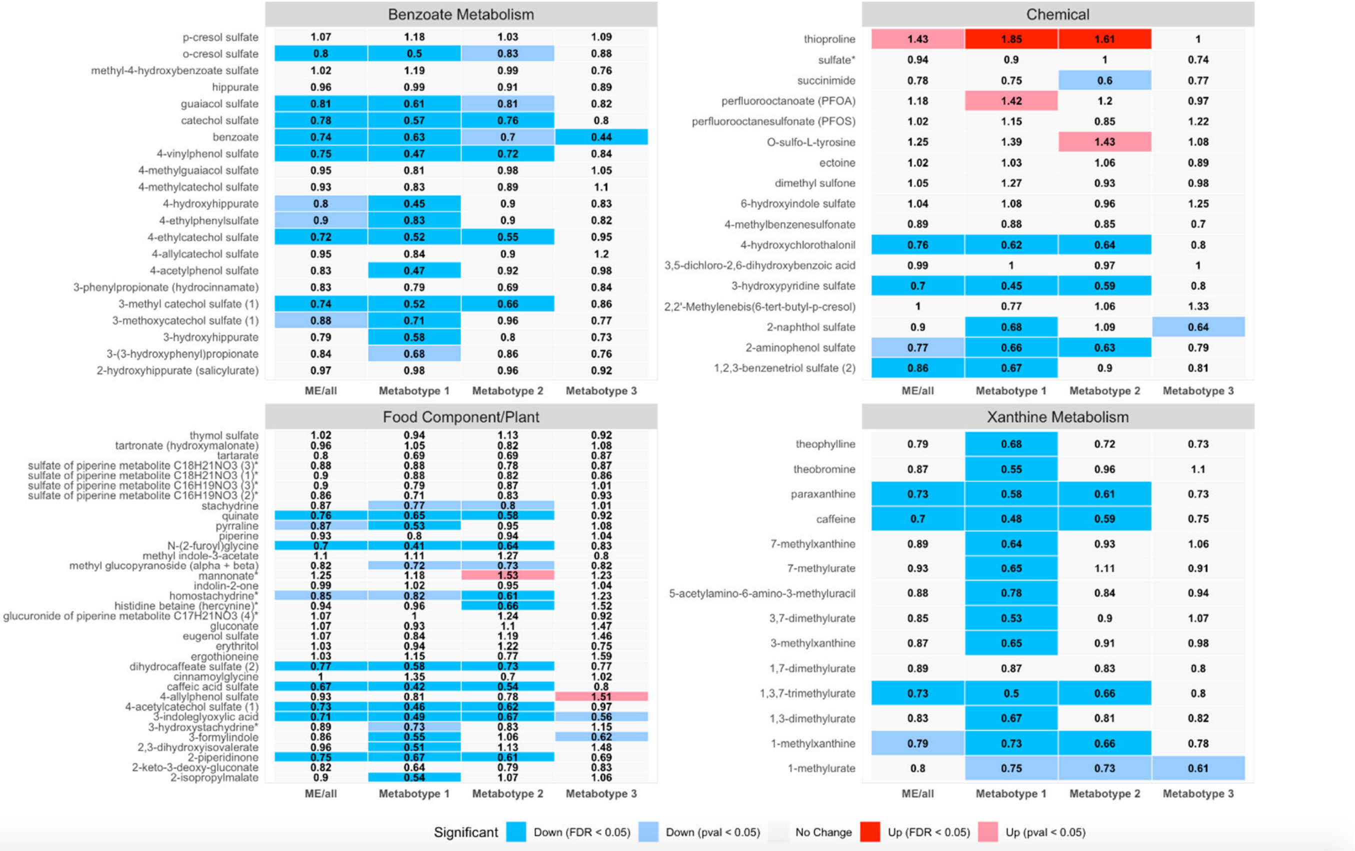

Figure 4: Food components: Heatmap with fold change values relative to healthy controls of xenobiotics (excluding drug components) originating from food consumption. Significant differences relative to healthy controls are color coded with red (increased), blue (decreased), with light (p < 0.05) or dark (FDR < 0.05) shades, and gray (no significant change).

6. References

1. Schrimpe-Rutledge AC, Codreanu SG, Sherrod SD, and McLean JA. Untargeted Metabolomics Strategies—Challenges and Emerging Directions. *Journal of The American Society for Mass Spectrometry.* 2016;27(12):1897-905.
2. Fluge O, Mella O, Bruland O, Risa K, Dyrstad SE, Alme K, et al. Metabolic profiling indicates impaired pyruvate dehydrogenase function in myalgic encephalopathy/chronic fatigue syndrome. *JCI Insight.* 2016;1(21):e89376.
3. Mora S. Nonfasting for Routine Lipid Testing: From Evidence to Action. *JAMA internal medicine.* 2016;176(7):1005-6.
4. Langsted A, and Nordestgaard BG. Nonfasting versus fasting lipid profile for cardiovascular risk prediction. *Pathology.* 2018.
5. Germain A, Barupal DK, Levine SM, and Hanson MR. Comprehensive Circulatory Metabolomics in ME/CFS Reveals Disrupted Metabolism of Acyl Lipids and Steroids. *Metabolites.* 2020;10(1):34.
6. Germain A, Ruppert D, Levine S, and Hanson M. Prospective Biomarkers from Plasma Metabolomics of Myalgic Encephalomyelitis/Chronic Fatigue Syndrome Implicate Redox Imbalance in Disease Symptomatology. *Metabolites.* 2018;8(4):90.
7. Germain A, Ruppert D, Levine SM, and Hanson MR. Metabolic profiling of a myalgic encephalomyelitis/chronic fatigue syndrome discovery cohort reveals disturbances in fatty acid and lipid metabolism. *Molecular BioSystems.* 2017;13(2):371-9.
8. Naviaux RK, Naviaux JC, Li K, Bright AT, Alaynick WA, Wang L, et al. Metabolic features of chronic fatigue syndrome. *Proceedings of the National Academy of Sciences of the United States of America.* 2016;113(37):E5472-80.
9. Nagy-Szakal D, Barupal DK, Lee B, Che X, Williams BL, Kahn EJR, et al. Insights into myalgic encephalomyelitis/chronic fatigue syndrome phenotypes through comprehensive metabolomics. *Scientific Reports.* 2018;8(1).
10. Tadesse MG, Sha N, and Vannucci M. Bayesian Variable Selection in Clustering High-Dimensional Data. *Journal of the American Statistical Association.* 2005;100(470):602-17.
11. Kim S, Tadesse MG, and Vannucci M. Variable selection in clustering via Dirichlet process mixture models. *Biometrika.* 2006;93(4):877-93.
