# Supplementary material for: A map of metabolic phenotypes in patients with myalgic encephalomyelitis/chronic fatigue syndrome
Source: JCI Insight. 2021 Aug 23;6(16):e149217. doi: 10.1172/jci.insight.149217 (PMC8409979; doi:10.1172/jci.insight.149217)
Supplement: Supplemental data 2 [file jciinsight-6-149217-s260.pdf]

Supplemental Data 2. Clinical blood biochemistry data of the ME/CFS metabotype subsets, per gender.

		ME/CFS		ME-M1		ME-M2		ME-M3	
**FEMALE**	**Unit**	**Mean ± SD**	**N**	**Mean ± SD**	**N**	**Mean ± SD**	**N**	**Mean ± SD**	**N**
**HEMOGLOBIN**	g/dL	13.78 ± 0.91	43	13.85 ± 0.96	18	13.55 ± 0.78	17	14.1 ± 1.06	8
**ALBUMIN**	g/L	45.60 ± 3.28	53	**47.4 ± 3.08**[a,b]	20	**44.36 ± 3.30**[a]	25	**45.00 ± 1.30**[b]	8
**FOLATE**	nmol/L	21.68 ± 10.05	59	**26.05 ± 10.46**[a]	21	**17.76 ± 9.07**[a]	28	23.50 ± 8.04	10
**GLUCOSE**	mmol/L	5.12 ± 0.82	55	5.04 ± 0.52	21	5.16 ± 1.05	25	5.20 ± 0.76	9
**CHOLESTEROL**	mmol/L	4.99 ± 0.84	60	4.90 ± 1.06	23	5.00 ± 0.68	28	5.20 ± 0.67	9
**HDL**	mmol/L	1.50 ± 0.36	60	1.60 ± 0.37	23	1.40 ± 0.35	28	1.54 ± 0.25	9
**LDL**	mmol/L	3.21 ± 0.86	46	3.19 ± 1.01	18	3.1 ± 0.74	21	3.57 ± 0.78	7
**TG**	mmol/L	1.41 ± 0.92	38	**0.98 ± 0.35**[a]	12	**1.72 ± 1.08**[a]	22	0.94 ± 0.12	4
**NEFA**	mmol/L	0.39 ± 0.30	57	**0.66 ± 0.28**[a,b]	20	**0.23 ± 0.18**[a]	27	**0.26 ± 0.17**[b]	10
**TSH**	mIU/L	2.12 ± 1.01	52	1.98 ± 0.98	20	2.23 ± 1.15	23	2.16 ± 0.70	9
**FREE T4**	pmol/L	16.17 ± 3.16	52	16.17 ± 2.44	20	15.96 ± 4.08	23	16.70 ± 1.86	9
**ACTH**	pmol/L	4.47 ± 2.87	52	**3.56 ± 1.33**[a]	20	**5.17 ± 3.05**[a]	23	4.68 ± 4.39	9
**CORTISOL (MORNING)**	nmol/L	405 ± 274.4	52	**275.2 ± 116.5**[a]	20	**528.0 ± 349.3**[a]	23	379.3 ± 161.2	9

		ME/CFS		ME-M1		ME-M2		ME-M3	
**MALE**	**Unit**	**Mean ± SD**	**N**	**Mean ± SD**	**N**	**Mean ± SD**	**N**	**Mean ± SD**	**N**
**HEMOGLOBIN**	g/dL	15.78 ± 1.00	11	16.12 ± 0.83	5	15.80 ± 0.93	4	14.90 ± 1.56	2
**ALBUMIN**	g/L	47.50 ± 2.25	16	47.29 ± 2.93	7	47.43 ± 1.72	7	48.50 ± 2.12	2
**FOLATE**	nmol/L	17.60 ± 9.83	18	19.39 ± 12.46	7	16.70 ± 9.07	9	15.40 ± 3.96	2
**GLUCOSE**	mmol/L	5.64 ± 0.84	16	5.81 ± 1.01	7	**5.24 ± 0.49**[c]	7	**6.40 ± 0.71**[c]	2
**CHOLESTEROL**	mmol/L	5.09 ± 1.26	18	**4.64 ± 0.66**[b]	7	5.00 ± 1.37	9	**7.05 ± 0.49**[b]	2
**HDL**	mmol/L	1.23 ± 0.26	18	1.23 ± 0.15	7	1.26 ± 0.34	9	1.10 ± 0.28	2
**LDL**	mmol/L	3.45 ± 1.05	17	**3.11 ± 0.72**[b]	7	**3.25 ± 0.91**[c]	8	**5.40 ± 0.14**[b,c]	2
**TG**	mmol/L	1.39 ± 0.70	10	1.40 ± 0.74	3	1.38 ± 0.81	6	1.42 ± 0.00	1
**NEFA**	mmol/L	0.36 ± 0.28	18	**0.63 ± 0.27**[a]	7	**0.16 ± 0.08**[a]	9	0.29 ± 0.21	2
**TSH**	mIU/L	2.22 ± 1.39	14	1.98 ± 1.82	6	2.43 ± 1.17	6	2.31 ± 1.12	2
**FREE T4**	pmol/L	17.07 ± 1.90	14	16.68 ± 2.29	6	17.67 ± 1.51	6	16.45 ± 2.33	2
**ACTH**	pmol/L	5.89 ± 2.78	14	6.35 ± 4.10	6	5.50 ± 1.27	6	5.70 ± 2.40	2
**CORTISOL (MORNING)**	nmol/L	420.79 ± 145.58	14	390.50 ± 133.05	6	419.33 ± 130.36	6	516.00 ± 280.01	2

[a] $p<0.05$, ME-M1 *vs* ME-M2, Welch's test.

[b] $p<0.05$, ME-M1 *vs* ME-M3, Welch's test.

[c] $p<0.05$, ME-M2 *vs* ME-M3, Welch's test.
