# Supplementary material for: A map of metabolic phenotypes in patients with myalgic encephalomyelitis/chronic fatigue syndrome
Source: JCI Insight. 2021 Aug 23;6(16):e149217. doi: 10.1172/jci.insight.149217 (PMC8409979; doi:10.1172/jci.insight.149217)
Supplement: Supplemental data 3 [file jciinsight-6-149217-s261.pdf]

Supplemental Data 3. Associations between uniform metabolite changes and patient characteristics.

Correlation analysis was performed to evaluate potential associations between the uniformly affected metabolites and patient characteristics at ME/CFS group level. As expected, both age and BMI positively associated with increased corticosteroid metabolites levels. However, these corticosteroids did not correlate with reduced Mean steps/24h or SF-36-PF. Increases in three metabolites (dihomo-linolenate 20:3n3 or n6, 5-methylthioadenosine MTA, 3-3-amino-3-carboxypropyluridine) associated with both increased BMI and reduced Mean steps/24h. Lowered concentration of two metabolites (beta-sitosterol and carotene diol 2) associated with both lowered BMI and reduced Mean steps/24h. Apart from this the metabolite correlation patterns of the two physical function scores appeared unrelated from those of age and BMI. Interestingly, the compounds that were either uniformly lowered or enriched in ME/CFS patients, mainly presented consistent associations with reduced function scores, and not age and BMI.

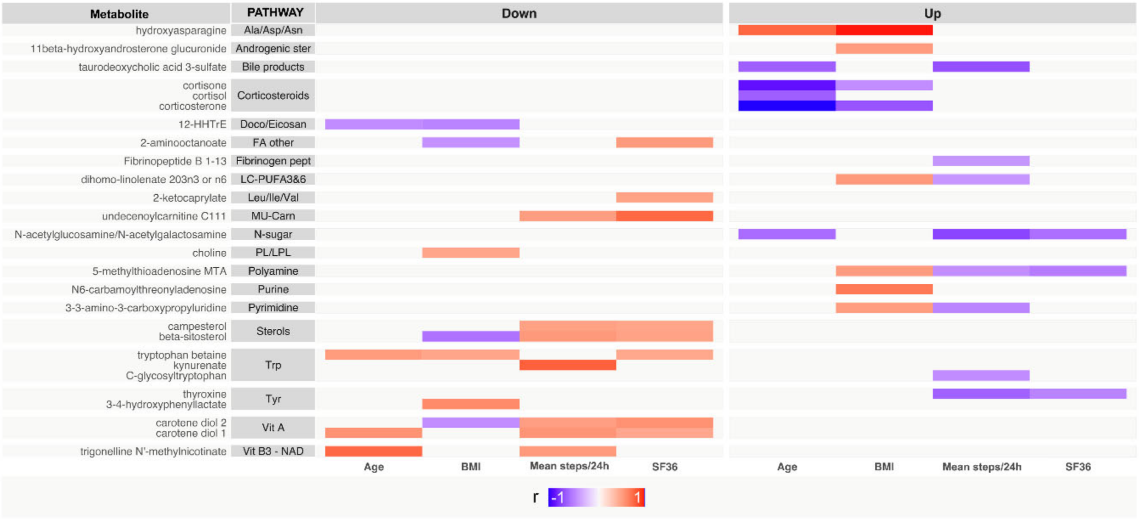

Supplementary Figure: Correlation analysis between uniform metabolites and key patient characteristics. The serum concentrations of metabolites presenting uniform change across the metabotypes were correlated with patient age, BMI, Mean steps/24h and SF-36-PF (SF36). Only metabolites exhibiting statistically significant correlation with one or more of the patient characteristics are shown (Spearman, $p < 0.05$). Metabolites that had low serum level in the ME/CFS group relative to the HC subject group are shown in the left panel, and those that were elevated in the right panel. Significant correlations are colored according to the Spearman correlation coefficient (rho, r), as indicated.
